# Implementing a digitally enabled wound model of care to improve chronic wound outcomes in seven health services in Australia: study protocol for a multisite, hybrid type 2, two-phase effectiveness-implementation trial (the ENABLE study)

**DOI:** 10.1136/bmjopen-2026-123987

**Published:** 2026-11-03

**Authors:** Michelle Barakat-Johnson, Michelle Lai, Andrew Wilson, Martin Howell, John Stephenson, Josephine Chow, Stephen M Twigg, Kate White, Astrid Frotjold, Catherine Leahy, Melissa T Baysari

**Affiliations:** 1 Susan Wakil School of Nursing and Midwifery, Faculty of Medicine and Health, The University of Sydney, Sydney, New South Wales, Australia; 2 Tissue Repair Institute, Sydney Local Health District, Camperdown, New South Wales, Australia; 3 School of Human and Health Sciences, University of Huddersfield, Huddersfield, UK; 4 School of Public Health, Faculty of Medicine and Health, The University of Sydney, Sydney, New South Wales, Australia; 5 South Western Sydney Local Health District, Sydney, New South Wales, Australia; 6 South Western Sydney Nursing and Midwifery Research Alliance, Sydney, New South Wales, Australia; 7 School of Medicine, The University of Sydney, Sydney, New South Wales, Australia; 8 Department of Endocrinology, Royal Prince Alfred Hospital, Sydney, New South Wales, Australia; 9 Cancer Care Research Unit, Faculty of Medicine and Health, The University of Sydney, Sydney, New South Wales, Australia; 10 Quality Clinical Safety and Nursing Directorate, Western New South Wales Local Health District, Orange, New South Wales, Australia; 11 School of Nursing, Paramedicine and Healthcare Sciences, Charles Sturt University, Bathurst, New South Wales, Australia

**Keywords:** Artificial Intelligence, Pragmatic Clinical Trial, eHealth, Nursing research, WOUND MANAGEMENT, Health Services

## Abstract

**Introduction:**

Chronic wounds place a substantial clinical, psychosocial and economic burden on individuals, health systems and communities. Fragmented and uncoordinated wound care practices lead to inefficiencies and variations in practice, delayed access to specialists and poor patient outcomes. While digital and artificial intelligence (AI) wound care technologies offer promising solutions, robust multisite effectiveness-implementation trials of these technologies in diverse real-world healthcare contexts are limited. This multisite study aims to evaluate the implementation and effectiveness of a digital model of wound care across seven metropolitan, regional, rural, and remote health services in Australia.

**Methods and analysis:**

The ENABLE study is a hybrid type 2, two-phase effectiveness-implementation trial of a digitally enabled model of wound care. Phase 1 is guided by the Consolidated Framework for Implementation Research and involves the co-development of tailored implementation strategies informed by patient and clinician interviews and focus groups to identify the barriers and facilitators to implementation across seven diverse clusters including primary care, community nursing, residential aged care and hospital inpatient and outpatient settings. In phase 2, the clinical effectiveness of the model is evaluated using a stepped-wedge cluster randomised controlled trial (SW-CRT) involving approximately 750 patients across seven clusters along with a mixed-methods evaluation and an economic analysis. The primary outcome is the proportion of patients admitted to hospital with a chronic or complex wound diagnosis. Secondary outcomes include wound size reduction, length of hospital stay, health-related quality of life, patient and clinician experiences, implementation outcomes (acceptability, appropriateness, feasibility, adoption, sustainability and fidelity) and cost-effectiveness.

**Ethics and dissemination:**

Ethical approval was obtained from the Sydney Local Health District Human Research Ethics Committee (protocol V.1.0; protocol no.: X24-0360; ethics identifier: 2024/ETH02394; approved 21 March 2025; current version V.7.0, 10 February 2026). All participants will provide written informed consent prior to participation. The results will be disseminated through peer-reviewed publications, presentations to conferences and health governing agencies and stakeholder educational activities. Clinical results will be reported in line with the Consolidated Standards of Reporting Trials (CONSORT) (SW-CRT extension) and Standards for Reporting Implementation Studies and Consolidated Criteria for Reporting Qualitative Research, and economic evaluation will be reported in line with the Consolidated Health Economic Evaluation Reporting Standards (CHEERS).

**Registration:**

Registered 2 June 2025 with the Australian New Zealand Clinical Trials Registry (ACTRN12625000562471).

**STRENGTHS AND LIMITATIONS OF THIS STUDY**

- The Consolidated Framework for Implementation Research is used to guide the assessment of barriers and facilitators, the co-development of tailored implementation strategies and the mixed-methods evaluation of outcomes.
- The stepped-wedge cluster randomised controlled trial design enables the rigorous evaluation of clinical effectiveness while enabling a phased, pragmatic implementation across diverse real-world healthcare settings, including primary care (general practice), community nursing, residential aged care and hospital settings. This design is particularly appropriate for testing whole-of-community interventions that are anticipated to deliver a net benefit and for which it would be ethically, socially or practically unacceptable to withhold through randomisation.
- Because the implementation of the ENABLE model represents a system-level change to usual care, blinding of clinicians and patients to intervention exposure is not feasible, potentially introducing performance bias.
- Concealment of the crossover date between usual care and the intervention is not possible because of implementation requirements, although the randomisation of clusters minimises selection bias.
- Variations in digital infrastructure, workforce capacity and readiness for AI-enabled tools across metropolitan, regional, rural, remote and very remote sites may influence implementation fidelity and the generalisability of findings.

## Introduction

 Chronic wounds place a substantial and increasing burden on individuals, health systems and communities.^1 2^ People living with chronic wounds experience prolonged hospital stays and unplanned hospital readmissions, delayed healing, higher infection risk^3^ and reduced capacity for self-care, diminishing their health-related quality of life (HRQOL).^1 4^ Patients commonly report pain, discomfort, embarrassment, low self-esteem, frustration and depression along with substantial out-of-pocket costs for general practitioner (GP) and specialist consultations.^5–7^

At a health system level, the challenges of uncoordinated and fragmented wound care continue to drive inefficiencies and poor outcomes.^8 9^ Patients frequently face long delays in accessing specialist wound care, extended gaps between clinical appointments,^7^ subjective and inconsistent wound assessment and inadequate communication across services.^10 11^ These systemic constraints contribute to wound deterioration, longer hospital stays, a higher number of readmissions and avoidable morbidity and mortality.^1^ Practitioners also face challenges delivering quality care because of poorly coordinated services, low-quality images and limited or inequitable access to resources, high-quality wound dressings, expert support and integrated data systems.^10 11^

Systematic reviews have consistently demonstrated the substantial clinical, psychosocial and economic burden associated with chronic wounds and the marked variations in care quality and outcomes across settings.^12 13^ In particular, patients with chronic wounds tend to have poor HRQOL.^13^ The economic burden of chronic wounds is also considerable. ^2 14^An Australian study of wound care costs demonstrated substantial treatment costs across hospitals, residential aged care, general practice and community settings, highlighting the significant resource burden associated with wound management.^2 14^ Similarly, in the USA, chronic wound care brings a substantial financial burden, with total Medicare costs estimated at US$28.1–96.8 billion annually,^15^ highlighting the unsustainability of current models.

Early digitally enabled wound care models such as Sydney Virtual Hospital’s (previously known as RPA Virtual Hospital) Wound Care Command Centre^16^ have led to promising improvements in patient access to and engagement with specialist expertise, timeliness of care and cost-effectiveness. However, early evaluations of these models have been mostly conducted in single health districts. Therefore, evidence on the real-world effectiveness, scalability and system-level outcomes of digitally enabled wound models implemented in multiple services and jurisdictions is limited.^17^ Given the profound clinical, economic and human burden associated with chronic wounds and the promising outcomes of early implementations,^18 19^ there is a clear need to evaluate the broader integration, scalability and outcomes of digital wound care models such as the Wound Care Command Centre.^18^ Importantly, there is limited evidence on the implementation and effectiveness of this model across diverse metropolitan, regional, rural, remote and very remote settings using a theory-informed implementation framework.

This paper presents a protocol for the ENABLE study (‘Improving patient outcomes and reducing the hidden burden of chronic wounds: a digitally enabled wound model of care’), a hybrid type 2 two-phase effectiveness-implementation study conducted across seven clusters in Australia. The ENABLE study is underpinned by partnerships between policymakers, health service managers, clinicians and researchers across participating clusters. The research team includes nationally and internationally recognised clinical, executive, policy and research leaders with extensive expertise in wound care, digital health, service redesign and translational research. These partnerships are central to the co-design, implementation and evaluation of the ENABLE model and are intended to support scalability, sustainability and real-world outcomes beyond the trial period.

### Rationale

The ENABLE model, a digitally enabled model of wound care, addresses a critical need in chronic wound care by integrating artificial intelligence (AI)-driven wound assessment with specialist clinical oversight to provide standardised, evidence-based care. Preliminary work in one health district has already demonstrated the feasibility and acceptability of this approach. Specifically, three studies^16 18 19^ were conducted to evaluate the person-centred, nurse-led Wound Care Command Centre, which provides digital wound assessments and treatment using the Tissue Analytics digital app and enables secure clinician-patient messaging, demonstrating its strong acceptability among both patients and clinicians. The results of these early studies were promising. Patients received an average of one service occasion every 4.4 days (vs every 13.4 days in standard care), while the median response time to patient queries was 1.5 hours.^18^ Clinicians reported improved patient engagement, enhanced adherence to treatment plans, improved ability to monitor and track wounds and rapid access to expert wound advice. Patients reported high satisfaction with their wound care. Of the 51 participants in one study, 84.1% found the digital wound app easy to use and 86.4% would recommend it for wound care management.^18^ Both patient and clinician groups strongly advocated for broader implementation of the model. Subsequently, the Wound Care Command Centre was expanded and launched in 2023 as part of a hybrid hospital (Sydney Virtual Hospital, previously RPA Virtual Hospital), providing an opportunity to evaluate the scalability and real-world implementation of this digitally enabled model of care across multiple services and settings.

The implementation strategy is guided by the Consolidated Framework for Implementation Research (CFIR)^20^ to ensure cluster-specific tailoring and the successful adoption of the model across diverse healthcare settings, including metropolitan, regional, rural, remote and very remote sites. We will identify barriers and facilitators, map usual care practices and co-develop facilitation strategies to optimise uptake and sustainability. By combining a digitally enabled intervention with a structured, theory-informed implementation approach, ENABLE is expected to enhance assessment timeliness, treatment standardisation, patient engagement and service efficiencies in chronic wound management.

### Study aims and objectives

The ENABLE study aims to evaluate the implementation and effectiveness of a digital model of wound care across diverse healthcare settings in Australia, including primary care, community nursing, hospital inpatient and outpatient settings and residential aged care facilities. The overarching objective of the ENABLE model is to provide timely access to specialised wound care for patients with chronic wounds and improve both patient and health system outcomes.

We will assess whether this complex digital intervention can be successfully embedded into routine practice and whether it leads to improved clinical and service outcomes. Specifically, the objectives of the ENABLE study are as follows:

1. Evaluate the implementation of ENABLE: using the CFIR as an implementation science framework and in partnership with clinical and organisational partners, evaluate implementation outcomes, including the adoption, feasibility, fidelity, acceptability, appropriateness and sustainability of the model.
2. Evaluate the effectiveness of ENABLE for patient and health service outcomes, including hospitalisations, chronic or complex wound admissions, length of hospital stay, wound healing and HRQOL.
3. Evaluate the cost-effectiveness of the ENABLE model of care compared with standard wound care to demonstrate the economic value of a digitally enabled approach.

## Methods and analysis

This protocol has been developed in accordance with the Standard Protocol Items: Recommendations for Interventional Trials (SPIRIT)^21^ (see online supplemental appendix 1).

### Governance and organisation

A key strength of ENABLE is its governance structure (see figure 1), which comprises a project steering committee, a clinical expert and consumer group (including a consumer representative, clinicians and implementation partners at participating sites) and a translation consortium, which includes Wounds Australia, the national peak body for chronic wound advocacy, education and awareness, and two key New South Wales (NSW) government stakeholders: eHealth NSW, a digital centre of excellence that plans, implements and supports information and communication technology and digital capabilities across the state of NSW and the Agency for Clinical Innovation, which works alongside clinicians, consumers and healthcare services to support the design, evaluation and adoption of innovative, evidence-informed approaches to healthcare delivery and clinical practice improvement.

**Figure 1:**
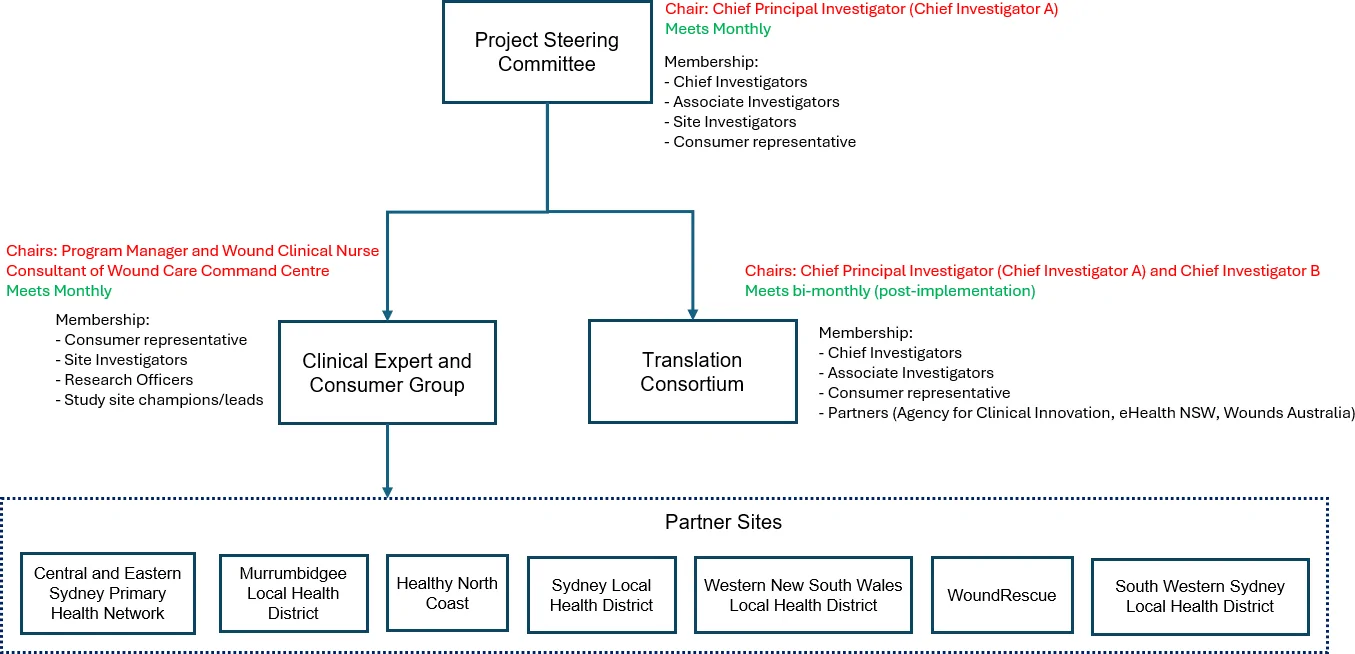
Project governance. NSW, New South Wales.

The project steering committee will oversee the study design and operationalisation according to the study protocol, ensure that risks are minimised, disseminate the results, identify research translation opportunities in consultation with the translation consortium and provide feedback and lessons learnt. The clinical expert and consumer group, comprising a consumer representative, site investigators, research officers and local champions, will provide clinical expertise, oversee clinical aspects (eg, screening patients, supervising staff and monitoring adherence to the protocol) and communicate with the project steering committee. The translation consortium includes policymakers, policy partners, a consumer representative and leaders and clinical representatives from partner sites. The consortium will report to the project steering committee and devise and roll out the dissemination strategy.

### Patient and public involvement

In phase 1 (co-development phase), tailored implementation strategies will be co-developed with patients and clinicians from our participating sites to facilitate the local uptake and sustainability of the ENABLE model. We will also conduct patient interviews and clinician focus groups and interviews to inform phase 2, where we will test the clinical effectiveness and cost-effectiveness of ENABLE.

A dedicated consumer representative with lived experience who has reviewed the protocol and patient-facing study documents will work with practice partners and investigators to provide insights on behalf of patients. The consumer representative will provide a lived experience perspective to ensure the intervention remains relevant to patients. They will also contribute to the clinical expert and consumer group and translation consortium, building on their previous contributions to projects that have informed the current study.

### Context and settings

The ENABLE model of wound care will be implemented across a diverse range of healthcare settings, including primary care (general practice), residential aged care, hospital inpatient and outpatient and community care settings in metropolitan, regional, rural, remote and very remote NSW. It will be targeted at all chronic skin wound types and aetiologies. The intervention will be situated in a complex and heterogeneous health system in which wound care delivery varies substantially according to funding model (federal, state or private), geography, service configuration, workforce capacity and access to specialist expertise. These contextual differences contribute to the well-documented inequities in access to timely specialist wound care, particularly outside of metropolitan centres.^22 23^

#### Participating sites

There are seven clusters, each comprising one or more sites across the following care settings: primary care (GP practices), community (community nursing), residential aged care, acute in-patient care, and outpatient care. (table 1). Study clusters will vary in terms of size, staff (GPs, practice nurses, community and hospital-based nurses and allied health professionals), availability of wound care specialists, digital infrastructure and patient populations (table 2). For partner services comprising multiple facilities, a subset of sites has been selected for participation on the basis of a structured readiness assessment covering feasibility of trial procedures, workforce capacity to deliver the model, digital infrastructure and executive and clinical leadership support for implementation. Table 1 describes each cluster and table 2 shows the number of participating sites for each partner service and the staffing mix.

**Table 1:** Study clusters

Cluster		Description
1	Sydney LHD	A large, highly complex metropolitan LHD servicing a high volume of diverse patients with 2.6 million outpatients, 180 000 ED presentations and more than 160 000 hospital admissions annually. Digital infrastructure is advanced, encompassing robotic surgery and a dedicated digital wound app. Sydney LHD was home to the first virtual health service in NSW (Sydney Virtual Hospital), established just before COVID-19. Building on this, the nurse-led Wound Care Command Centre was implemented in 2023 as a hybrid wound service, integrating virtual and in-person care to meet the demands of a high-acuity, high-volume clinical environment.^44^
2	South Western Sydney LHD	One of the largest, fastest growing and most culturally diverse metropolitan LHDs in NSW, with six acute public hospitals and 14 major community health centres providing prevention, early intervention, community care, palliative care and rehabilitation services. The LHD has more than 1.1 million residents across an area of 6243 km^2^. Digital infrastructure is well established, including district-wide electronic medical records and virtual care capabilities, with increasing adoption of digital wound technologies.^45^
3	Central and Eastern Sydney PHN	A federally funded metropolitan PHN with the key objective of increasing the efficiency and effectiveness of medical services, particularly for those at risk of poor health outcomes. The PHN is the second largest in Australia, servicing 1.5 million people across an area of 587 km². It works directly with key players, including GPs, allied health practitioners, nurses, secondary care providers, LHDs and specialty health networks.^46^
4	Western NSW LHD	A large rural and remote LHD spanning 246 676 km² and servicing approximately 280 000 people across 38 inpatient facilities, 25 multipurpose services with residential aged care and 50 community health centres.^47^ The district faces ongoing challenges in access, workforce distribution and service delivery and services the highest proportion (11%) of Aboriginal and Torres Strait Islanders in NSW. A nurse-led virtual wound consultancy service^22^ was established in 2018 to address the lack of wound specialists in inpatient, outpatient and residential aged care settings. The service connects local clinicians with wound specialists via virtual consultations for assessment, management and ongoing clinical support, removing the need for patients to travel long distances. It also builds local capability through education, case reviews and guidance on complex wound management.
5	WoundRescue	A network of private advanced wound consultation services for patients living with chronic or complex wounds in Australia. The service consults with approximately 15 000 patients per year, most of whom live in residential aged care (45–360 beds).^48^
6	Healthy North Coast	A federally funded regional and rural PHN that collaborates with two LHDs, GPs and allied health providers to strengthen the overall healthcare system. Covers roughly 32 047 km^2^ and supports over 520 000 residents, many of whom are older adults and Aboriginal and Torres Strait Islander people. Its primary objective is to keep people well, particularly those with chronic physical and mental illness, and reduce pressure on the hospital system. To achieve this, it delivers national programs using a place-based approach, tailoring initiatives to meet local health needs.^49^
7	Murrumbidgee LHD	A regional LHD in southern NSW spanning 125 243 km² and provides hospital-based, community health and virtual care services to more than 250 000 people in rural, regional and remote communities. Its ~118 089 annual hospital admissions are characterised by a high proportion of Aboriginal and Torres Strait Islander people. The LHD partners with community organisations, primary care clinics, specialist networks and state and national agencies to improve outcomes, with services designed to be culturally safe, inclusive and responsive for culturally and linguistically diverse communities, including Aboriginal and Torres Strait Islanders, and people with complex needs.^50^

ED, emergency department; GP, general practitioner; LHD, local health district; NSW, New South Wales; PHN, primary health network.

**Table 2:** Participating sites and staffing profile in each cluster in the ENABLE study

Cluster		Site	Wound care staff
1	Sydney LHD	Two RACFs	CNCs, transitional nurse practitioners
2	South Western Sydney LHD	Four community nursing services	CNCs, clinical nurse specialists, registered nurses
3	Central and Eastern Sydney PHN	Two general practices	GPs, practice nurses
4	Western NSW LHD	Eight inpatient and outpatient facilities	CNCs, transitional nurse practitioners
5	WoundRescue	Two RACFs; one community nursing service	CNCs, clinical nurse specialists
6	Healthy North Coast	Two general practices; one RACF	General practices: GPs, practice nurses RACF: nurses
7	Murrumbidgee LHD	60 inpatient, outpatient and community nursing facilities	CNCs, clinical nurse specialists

Practice nurses provide support for patient care in general practices under the clinical governance of GPs. CNCs are advanced practice nurses providing specialist clinical leadership, consultations and referrals. Transitional nurse practitioners are advanced practice nurses in training to independently diagnose and prescribe. Only the services listed in the ‘Site’ column participating in the ENABLE trial.

CNC, clinical nurse consultant; GP, General practitioner; LHD, local health district; NSW, New South Wales; PHN, primary health network; RACF, residential aged care facility.

### Study design

The ENABLE study is a two-phase effectiveness-implementation study designed to evaluate both the clinical effectiveness of the ENABLE model of wound care and the success of its implementation across multiple healthcare settings. Clinical effectiveness will be assessed using a stepped-wedge cluster randomised controlled trial (SW-CRT), while implementation outcomes will be examined using quantitative (survey) and qualitative (interview and focus group) methods, with clusters randomised to the timing of crossovers and clinicians delivering the intervention. An economic evaluation is also planned.

Consistent with a hybrid type 2 design, the study addresses effectiveness and implementation aims concurrently, with implementation strategies intended to be practical and replicable and implementation outcomes specified a priori.^24^ All implementation outcomes in table 3 are treated as pre-specified implementation endpoints, assessed descriptively and thematically rather than through formal hypothesis testing, consistent with a hybrid type 2 design in which implementation and effectiveness aims are addressed concurrently.

**Table 3:** Implementation outcomes in the ENABLE study: definitions, data sources, measurement, timing and analysis

Outcome	Definition	Data source and measurement	Timing and analysis
Acceptability	Perception among clinicians and patients that the ENABLE model is agreeable and satisfactory	Clinician surveys; CFIR-guided clinician interviews and focus groups; patient interviews and patient-reported experience measure	Phase 2, during and at the end of each cluster’s intervention period; descriptive and thematic analysis
Appropriateness	Perceived fit and relevance of the model to each setting and to chronic wound care	CFIR-guided clinician interviews and focus groups	Phase 2, final 2 months of intervention period; thematic analysis mapped to CFIR
Feasibility	Extent to which the model can be delivered within existing workflows and resources	CFIR-guided clinician interviews and focus groups; implementation logs	Phase 2, during and at the end of intervention period; thematic analysis and descriptive summary
Adoption	Uptake of the model by clinicians and services	Proportion of eligible clinicians completing training and actively using the model; proportion of eligible patients managed through the model; training records, app data and medical records	Phase 2, monthly during each cluster’s intervention period; descriptive analysis by cluster and period
Fidelity	Delivery of the core components of the model as intended	App usage analytics; training completion records; wound consults	Phase 2 during intervention period; descriptive analysis and pre-specified sensitivity analysis
Sustainability	Continued delivery of the model beyond the trial period and perceived capacity to sustain it	Clinician interviews and focus groups; routinely collected app and service data on continued use for up to 6 months after site completion	End of phase 2 intervention period and up to 6 months post completion; thematic and descriptive analysis

Outcome definitions follow Proctor *et al.*

CFIR, Consolidated Framework for Implementation Research.

Across both phases, the CFIR will be used to inform the implementation and evaluation of ENABLE.^25^ The CFIR synthesises 19 implementation science theories to outline the constructs (intervention characteristics, outer and inner settings, participant characteristics and implementation process) necessary for the successful implementation of an intervention. It has been used extensively in the evaluation of policy and practice interventions, including digital health interventions.^26^ Guided by this framework, this study will employ mixed methods to systematically examine the barriers to and facilitators of the implementation of the ENABLE model of care.

The study uses a SW-CRT design comprising a control period of 5 months (first cluster commenced August 2025), a 1-month transition period during which no outcome data are collected (first cluster commenced January 2026) and a 6-month intervention period (first cluster commenced February 2026). Clusters were randomly allocated to one of seven sequences that determine the timing of transition from standard care to ENABLE. Clusters will transition from control to intervention in a staggered manner at approximately 1-month intervals, resulting in an incomplete stepped-wedge design that supports phased implementation while reducing data collection burden.^27 28^ Following trial set-up, the total study duration will be approximately 12–18 months. The participants assessed in each period may differ, reflecting an open cohort design rather than repeated measurements of the same participants. Figure 2 shows the sequence allocation, crossover timing and data collection periods.

**Figure 2:**
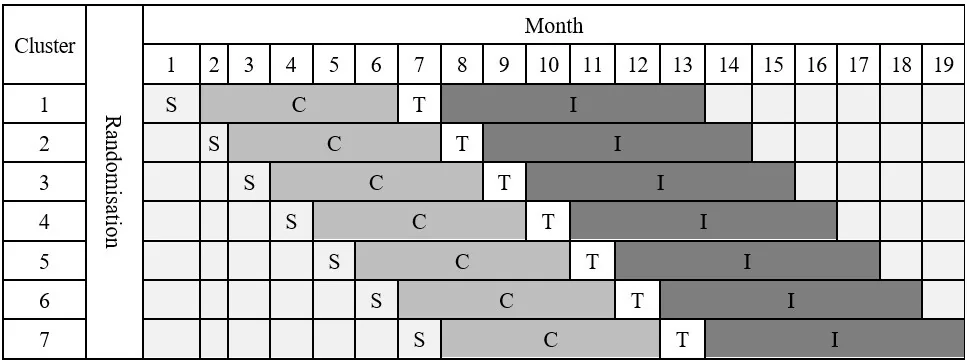
Stepped-wedge cluster randomised controlled trial schema for ENABLE trial. C, control period; I, intervention period; S, setup period; T, transition period.

This study complies with the National Clinical Trials Governance Framework,^29^ which supports the delivery and integration of high-quality clinical trials in routine hospital care to improve patient outcomes. The study protocol also aligns with Australian^30^ and NSW^31^ frameworks guiding the design, development and use of AI-enabled products and solutions. Figure 3 presents the timeline of activities, showing the study phases, approvals, implementation, data collection and analysis and governance activities across the 3-year period (September 2024–August 2027). Clinicians who are also investigators will implement the ENABLE intervention with the support of the study investigators. Activities are organised by quarter, with staggered cluster entry and overlapping governance structures throughout.

**Figure 3:**
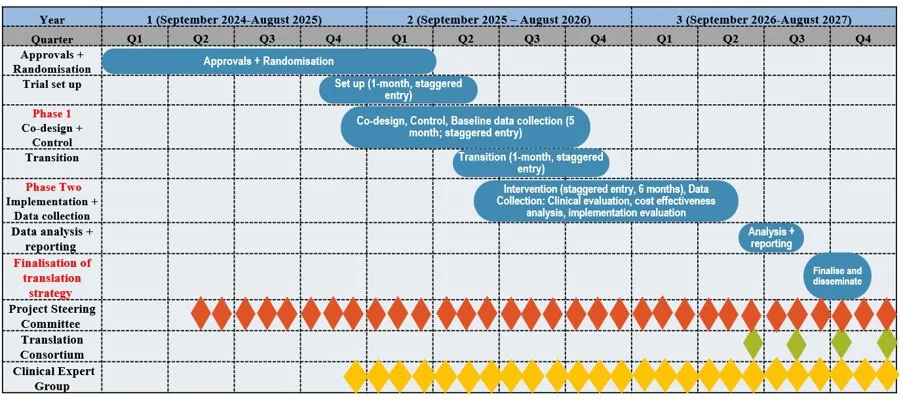
Timeline of activities.

### Recruitment and eligibility

#### Patient participants

Patients will be recruited for phases 1 and 2 of the study via a written informed consent process (see online supplemental appendices 2 and 3 for English-language participant information and consent forms).

The target population comprises adults (≥ 18 years) with chronic or complex wounds receiving care at one of the following participating services in primary care (GP practices), community (community nursing), residential aged care, acute in-patient care or outpatient care (see table 4). According to NSW Health value-based healthcare guidelines, a chronic or complex wound is one that has failed to heal within 30 days.^32^ Patients who present with superficial, rapidly healing wounds such as abrasions, blisters and superficial lacerations will be excluded. Treating clinicians will identify and approach eligible patients at the point of admission or from existing caseloads and confirm their eligibility prior to enrolment. Interested patients will be referred to the cluster research officer, who will provide the participant information sheet, answer questions and obtain written informed consent.

**Table 4:** Anticipated patient participant numbers

Cluster	Types of setting	Target no. patients
1	Residential aged care facility	60
2	Community nursing	100
3	General practice	60
4	Acute inpatient and outpatient	200
5	Residential aged care facility and community nursing	60
6	General practice and residential aged care facility	120
7	Acute inpatients, community nursing and outpatients	150

See ‘Sample size’ section on how expected patient participant numbers were determined.

Culturally and linguistically diverse and Aboriginal and Torres Strait Islander patients are expected to be among those meeting the eligibility criteria. Interpreters and Aboriginal liaison officers will support staff with the recruitment.

Following obtaining informed written consent, each enrolled participant will be allocated a unique study identification code. This code will be used on all study documents and datasets to ensure that personal identifiers are removed and participant confidentiality maintained. Any identifying information will be stored separately from the study data to ensure confidentiality.

#### Clinician participants

Clinicians will be recruited from participating hospital and primary care sites across both the control and the intervention periods. Recruitment will occur through sitewide email and staff meeting invitations to ensure an arm’s-length approach and minimise perceptions of coercion. Interested clinicians will contact the research officer directly, and will be provided with a participant information sheet and consent form (see online supplemental appendices 4 and 5), the opportunity to ask questions and time to consider participation prior to providing written informed consent.

Eligible clinicians will include nursing, medical and allied healthcare professionals involved in wound care, including practice nurses, frontline hospital enrolled nurses, registered nurses, nurse practitioners, clinical nurse consultants, GPs, podiatrists, occupational therapists and hospital-based medical practitioners. Clinicians must be employed at the participating study site and be involved in the assessment, consultation and ongoing management of patients with chronic or complex wounds.

Clinicians will be recruited as a cross-sectional sample from those delivering wound care at the time of each phase; the same individual may or may not participate in both phases. This reflects the open cohort design of the study, consistent with the approach used for patient recruitment.

### Intervention

The ENABLE model (see figure 4) is an integrative model of wound care that acts as a bridge between hospital, specialist and community care. It delivers person-centred wound management through a specialist multidisciplinary service, enabling timely access to expert advice for clinicians, patients and carers via both virtual and face-to-face consultations. Supported by a purpose-designed digital app approved by the Therapeutic Goods Administration^1^ (ARTG ID 374066) and integrated with patient electronic medical records, the model facilitates real-time clinical decision-making, secure communication and coordinated shared care across hospital and community settings. The app functions as a decision-support tool; all treatment decisions rest with the treating clinician. Where a clinician considers an app-generated measurement to be inaccurate, they may retrace the wound boundary and recapture the measurement. The study aligns with the Australian National Framework for the Assurance of Artificial Intelligence in Government and the NSW Artificial Intelligence Assessment Framework.^30 31^ Integrated data systems further support the continuity of care by linking wound data with broader clinical records, enabling progress monitoring and providing standardised measures to assess treatment effectiveness and patient outcomes. Patients will be reviewed weekly or as clinically required according to patient factors and wound complexity and healing progress. Moreover, wounds are not necessarily reviewed by the same clinician at each consult. Treatment is based on wound type, severity, comorbidities, availability of wound care products and patient compliance and acceptance.

**Figure 4:**
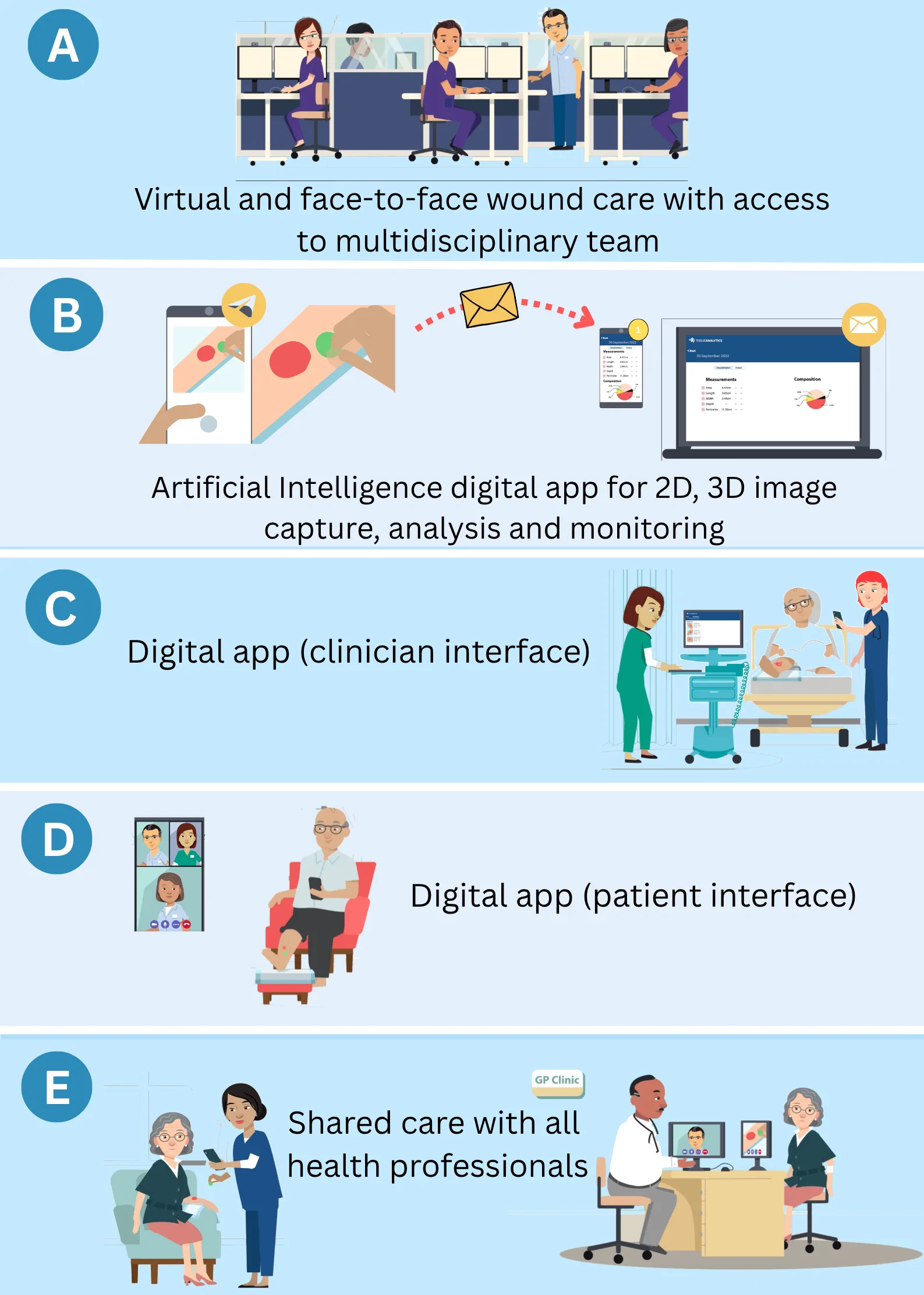
ENABLE model of care. (A) Wound Care Command Centre (core intervention delivery): virtual and face-to-face specialist wound care with access to onsite general practitioners and a multidisciplinary team if needed. (B) AI digital wound app (Tissue Analytics) for image capture, analysis, tracking and monitoring. (C) The digital app is used at the Command Centre and across hospitals and community to support clinicians in care delivery and decision-making. (D) The digital app supports patient engagement and self-monitoring; Tissue Analytics is optimal for its patient-facing features. (E) Shared care occurs between practitioners and specialists, supporting coordination across care teams. AI, artificial intelligence.

The core components of the ENABLE model, required at all sites, are: access to specialist wound review through the Wound Care Command Centre or equivalent service; digital wound assessment and monitoring using the Tissue Analytics app and documented shared care communication between the specialist service and the local treating team. Adaptable elements, which may be tailored to local context, include referral and escalation pathways, the mix of virtual and face-to-face review, the frequency of review (which is clinically determined), local workflow integration and the format of training and communication.

### Implementation strategy

The ENABLE implementation strategy is a CFIR-guided, co-designed, two-phase, multicomponent approach tailored to each partner cluster.

In phase 1, qualitative data from patient interviews and clinician focus groups and interviews will be used to understand usual care, identify barriers and facilitators and inform site-specific strategies. These strategies will be reviewed and operationalised by the clinical expert and consumer group. Strategies will most likely include clinician training on Tissue Analytics, workflow adaptations to support timely wound monitoring, structured communication channels such as newsletters and regular site meetings. Monitoring and feedback mechanisms will be used to track the adoption, fidelity and acceptability of ENABLE, with iterative refinements based on ongoing input from staff and patients.

Selection: candidate strategies are generated by mapping the CFIR-coded barriers and facilitators from the phase 1 qualitative analysis to strategies, which are then prioritised and operationalised for each cluster by the clinical expert and consumer group on the basis of feasibility, local fit and anticipated impact.

Delivery: implementation support is delivered by cluster research officers and site-based local champions, supported by the central project team and, for clinical practice questions, the Wound Care Command Centre specialists.

Recipients: implementation support is directed at the clinicians delivering wound care at each site (as listed in table 2) and their line managers, whose engagement is required for workflow change.

Documentation: all implementation activities (training sessions, site meetings, newsletters, feedback reports and any adaptations) are recorded prospectively in a structured implementation log maintained by each cluster research officer, which will be used in the process evaluation and fidelity assessment. Adaptations will be recorded including what was adapted, by whom, why and with what anticipated effect and will be reviewed by the clinical expert and consumer group to confirm that core components remain intact.

During phase 2, the strategies co-developed in phase 1 will be used to guide the staged rollout of ENABLE through the SW-CRT, supporting uptake across all study settings and promoting standardised, evidence-based care. Co-design, local adaptation, training, workflow integration and continuous feedback are aimed at ensuring the sustainable adoption, acceptability and effectiveness of ENABLE while addressing site-specific challenges.

Fidelity is defined as delivery of the core components of the ENABLE model as intended (specialist review via the Wound Care Command Centre or equivalent service, digital wound assessment using the Tissue Analytics app and documented shared-care communication).

It will be measured using app usage analytics (proportion of enrolled patients with wound assessments captured digitally), training completion records and wound consults under the new model of care in each cluster, conducted during the intervention period. Fidelity data will be reviewed by the clinical expert and consumer group, and project steering committee with feedback provided to sites as part of the monitoring and feedback strategy. In the effectiveness analyses, fidelity will be reported descriptively by cluster and used in a pre-specified sensitivity analysis restricted to clusters and periods meeting a minimum fidelity threshold, with the intention-to-treat analysis remaining primary. Qualitative interviews with clinicians and patients will further explore factors influencing fidelity.

Four strategies will be used to minimise inter-site heterogeneity. First, a strouctured site readiness assessment (digital infrastructure, workforce capacity and leadership support) will be completed for each site before enrolment, and sites within larger services were selected on this basis. Second, all sites will receive standardised training on the ENABLE model and the Tissue Analytics app, delivered by local champions appointed at each site to support uptake. Third, the phase 1 co-design process deliberately tailors implementation strategies to each cluster’s context while preserving the core components of the model, so that heterogeneity is addressed through planned adaptation. Finally, monthly cross-site meetings of the clinical expert and consumer group will provide a mechanism for identifying and responding to emerging site-level barriers during rollout. Contextual change will be monitored prospectively throughout implementation via the structured implementation log (recording changes in staffing, workflow or digital infrastructure at each site) together with CFIR-guided discussion of emerging contextual factors at the monthly cross-site meetings, providing a running record for use in the process evaluation.

Figure 5 summarises the full implementation logic model, integrating these strategies with the anticipated determinants, mechanisms and outcomes across all participating clusters.

**Figure 5:**
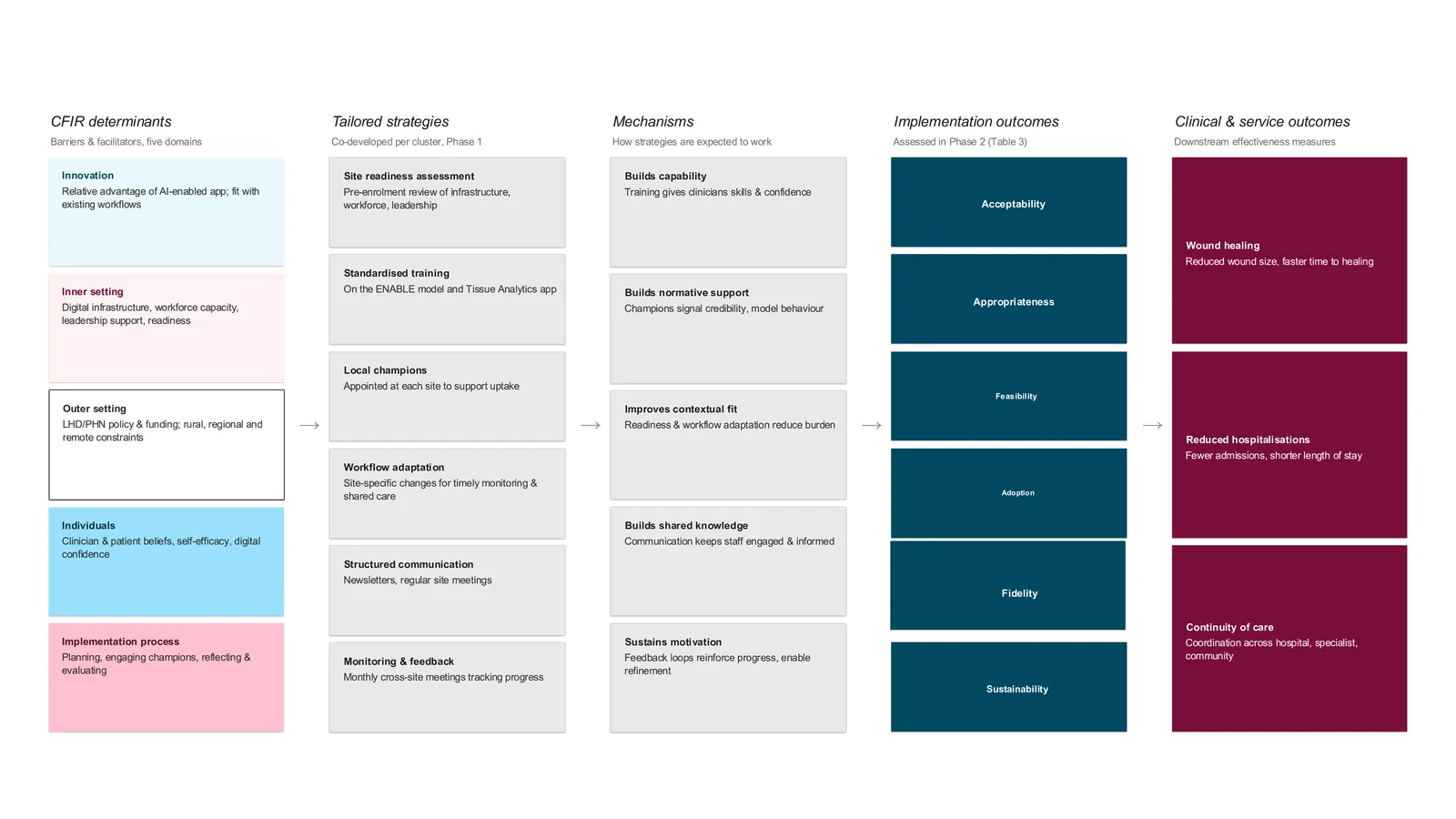
ENABLE implementation logic model. AI, artificial intelligence; CFIR, Consolidated Framework for Implementation Research; LHD, local health district; PHN, primary health network.

The model maps: (1) CFIR determinants across five domains (innovation, inner setting, outer setting, individuals and implementation process); (2) tailored implementation strategies selected to address them; (3) the mechanisms through which strategies are expected to operate; (4) anticipated implementation outcomes (acceptability, appropriateness, feasibility, adoption, fidelity and sustainability) and (5) downstream clinical and service outcomes (wound healing, reduced hospitalisations and improved continuity of care). This mapping reflects the hybrid type 2 design of the study, in which implementation strategies are specified a priori alongside effectiveness aims. Developed with reference to the CFIR outcomes,^33^ the hypothesised pathways in the logic model will be revisited and reported against in the trial’s primary effectiveness and process evaluation publications.

### Standard care

Standard wound care differs across study settings. In primary care settings (clusters 3 and 6), patients typically wait for scheduled appointments with GPs or nurses and have limited access to wound specialists. In community nursing services (clusters 2, 5 and 7), patients are typically followed up by a community nurse within 2 weeks of hospital discharge, with care plans reviewed at each visit and referral to specialist nurses for complex wounds when available. Patients at residential aged care facilities (clusters 1, 5 and 6) encounter heterogeneous wound management practices and variable access to external wound specialists. In rural and regional hospital (outpatient and inpatient) settings (clusters 4 and 7), optimal wound care is often constrained by limited specialist availability, workforce shortages and skill gaps.

### Outcomes

#### Primary outcome

The primary outcome of this study is the proportion of patients admitted to hospital with a chronic or complex wound (ie, a wound that has not healed within 30 days). This outcome will be assessed using routinely collected administrative and clinical data (from the patient’s medical record) and measured throughout the control and intervention periods, with comparisons made between standard care and intervention exposure periods. Participants will be followed up for a maximum of 90 days from the date of enrolment (or fewer if discharged from care or if they withdraw from the trial).

#### Secondary outcomes

1. Percentage reduction in wound size surface area, measured using the Tissue Analytics app. Wound size will be recorded at baseline and at weekly clinical reviews or documented assessments until healing, discharge or end of follow-up for up to 90 days from enrolment. Reduction in wound size at the time of discharge (baseline and up to 90 days from the date of enrolment) is a valid indicator of wound healing and treatment effectiveness.
2. Length of hospital stay, defined as the number of days from admission to discharge, obtained from administrative and medical records, extracted for each admission occurring within the 90-day follow-up period. Length of stay reflects healthcare utilisation and patient recovery efficiency.
3. Cost-effectiveness of ENABLE versus standard care based on estimates of healthcare costs and outcomes from patient-level and aggregated site-level data linked to primary, emergency and outpatient care and hospital admissions during phases 1 and 2.
4. Patients’ HRQOL measured using the EQ-5D-5L (EuroQol - 5 Dimensions - 5 Levels) at enrolment and at the final wound consultation (90 days following enrolment or discharge from the service, whichever occurs first).^34^ This will also enable the calculation of quality-adjusted life years (QALYs) for use in the cost-effectiveness analysis.
5. Patients’ care experiences using interviews and a patient-reported experience measure (online supplemental appendices 6 and 7), administered at the final wound consultation. Patient interviews will be conducted during the control period (phase 1) and after a minimum of 4 weeks of intervention exposure (phase 2) (online supplemental appendices 8 and 9).
6. Clinicians’ experiences of providing care in phases 1 and 2 via semi-structured interviews and focus groups, conducted pre-implementation (phase 1) and during the final 2 months of each cluster’s intervention period (phase 2).
7. Implementation outcomes, including how and why ENABLE was effective (acceptability, appropriateness, feasibility, adoption, fidelity and sustainability; definitions, data sources, measurement approaches and timing are provided in table 3) and the barriers and facilitators to successful implementation, which will be explored in the clinician interviews and focus groups (phase 2 only). Adoption will be measured as the proportion of eligible clinicians completing training and actively using the ENABLE model, and the proportion of eligible patients managed through the model during the intervention period, derived from training records and app and medical record data. Sustainability will be assessed in two ways: perceived sustainability, through clinician interviews and focus groups in the final 2 months of each cluster’s intervention period exploring intentions and perceived capacity to continue the model and the organisational conditions required and continued delivery, through routinely collected app and service data describing use of the model at each site for up to 6 months after site completion, reported descriptively.

### Adverse events (harms)

Serious adverse events, defined as hospitalisation from wound deterioration related to ENABLE, will be monitored and reviewed by an independent wound care expert. These events will be tabulated and reported with effect outcomes when determining study effectiveness outcomes. Any potential intervention-related events will be reported to the Human Research Ethics Committee (HREC).

### Sample size

#### Patient interviews and clinician focus groups and interviews (phases 1 and 2)

In each phase, approximately 35 patients and 40 clinicians will participate in interviews or focus groups. Recruitment will continue until thematic saturation is achieved, which is expected to occur after 30-40 interviews. Sample size calculations are based on previous experiences of data saturation in our pilot work on the digital wound model of care in the Sydney Local Health District (LHD).^16 18^

#### SW-CRT patient sample size (phases 2)

For an incomplete SW-CRT, seven clusters with an average of 99 patients per cluster will be sufficient to detect a 21.5% difference with a power of 83.7% between the proportion of patients in the control and intervention groups admitted to hospital with a chronic wound. The proportion of patients in the control group is estimated to be 36.5% based on the number of hospital admissions from 2013 to 2017.^9^ A medium effect size is assumed, corresponding to an assumed treatment proportion of 15% under the alternative hypothesis. No data are collected during certain periods, including the 1-month transition period (all clusters), with staggered entry points for each cluster, yielding a required sample size of 693 subjects before attrition.

A two-sided Wald *z*-test will be used. The intraclass correlation coefficient is assumed to be 0.02, with a significance level (alpha) of 0.05. We estimate an overall attrition of 7.5%. Therefore, we aim to recruit 750 patients in total, with an average of 107 per cluster.

Clusters were randomly allocated by the trial statistician in the presence of an independent witness to one of the predetermined sequences (see figure 2) using a simple randomisation process (ie, opaque sealed envelopes). Trial staff were not involved in and did not influence the randomisation procedure in any way. No constraints on randomisation or stratification were used in the randomisation process.

### Data collection

#### Patient and clinician interviews

Semistructured interviews and focus groups will be conducted with patients and clinicians in both phase 1 and phase 2 to explore their experiences of wound care and the implementation of the ENABLE model. Patients will be invited by the cluster research officer to be interviewed on their experiences with usual care (phase 1) and the ENABLE intervention (phase 2). Clinicians will participate in interviews or focus groups examining their current practices and barriers (phase 1) and model implementation outcomes, and facilitators and barriers (phase 2), guided by the CFIR.

The phase 2 qualitative data collection, implementation logs and quantitative implementation outcome data will together constitute a process evaluation. The full patient and clinician interview and focus group guides for both phases are provided in online supplemental appendices 8–11.

Data collection will occur in person or by videoconferencing or telephone. Patient interviews will run for 20–30 min, while clinician interviews and focus groups will run for 30–45 min. Clinicians will complete a brief demographic questionnaire prior to participation.

All sessions will be audiorecorded and transcribed in Microsoft Teams, with transcriptions reviewed for accuracy and de-identified prior to analysis. Phase 1 interviews will be conducted by the cluster research officer, while some phase 2 interviews may be conducted by a higher degree research student under supervision of the principal investigator.

#### Quantitative clinical data

Clinical outcomes of chronic and complex wounds will be routinely assessed by the treating clinical staff, independent of the research team. Research officers will prospectively collect data from electronic medical records and the Tissue Analytics app. Data collected will include patient demographics, wound characteristics, wound healing outcomes, relevant clinical variables and primary and secondary study outcomes.

#### Economic evaluation

Data will be collected from multiple sources to capture healthcare utilisation, clinical outcomes, costs and patients’ HRQOL. Patient-level demographic and clinical data (eg, age, gender and diagnosis), hospital admissions and wound healing outcomes will be extracted from medical records and the Tissue Analytics app. For participating LHDs (clusters 1, 2, 4 and 7), these data will be supplemented by linked administrative datasets, including Australian Refined Diagnosis-Related Groups, emergency department presentations and tier 2 outpatient service codes. These datasets will capture activity-based healthcare utilisation.

HRQOL will be collected using the EQ-5D-5L^34^ on enrolment and at the final wound consultation (90 days following enrolment or discharge from the service, whichever occurs first).

In addition, for eligible patients not enrolled in the trial, aggregated, non-identifiable cohort-level data will be collected from participating LHDs. These data will be used to characterise service-level activity, costs and outcomes before and after implementation.

### Data management

Electronic data will be stored securely on the following University of Sydney databases: (1) highly protected SharePoint (audio files and transcripts of patient and clinician interviews and focus groups and de-identified admission data), (2) research data store (de-identified Medicare Benefits Schedule and Pharmaceutical Benefits Scheme data) and (3) research electronic data capture (REDCap)^35 36^ (consent records, patient survey data, clinician demographic data and patient clinical audit data). Hardcopy survey data will be entered into REDCap by the cluster research officer.

Wound images captured in the Tissue Analytics app are stored within the app’s secure, encrypted environment and linked to the patient’s electronic medical record; images are not stored on clinicians’ personal devices. Access is role-based and restricted to the treating team and authorised study personnel. The platform is Therapeutic Goods Administration (TGA)-approved (ARTG ID 374066) and its deployment complies with NSW Health data security and privacy requirements, applicable privacy legislation and the NSW Artificial Intelligence Assessment Framework.^31^ Study data are held on University of Sydney secure systems with de-identification applied before analysis and data sharing.

Hardcopy consent forms, field notes and surveys will be securely stored in locked cabinets at each site, accessible only to authorised personnel, for 15 years following the trial. Electronic data will be entered from source documents by trained research officers using predefined validation rules to minimise error. Data quality will be supported through standardised procedures, routine monitoring and periodic review by the program manager, in accordance with good clinical practice (eg, ISO 14155:2026),^37^ with queries resolved in consultation with site teams. Given the nature of the intervention—a model of care involving access to a specialist wound service and a digital wound platform—a data safety monitoring board is not required. The investigators will, however, nominate an independent wound expert in Australia to perform a quarterly review of serious adverse events to determine whether they are attributable to the intervention, in which case they will notify the HREC.

### Data analysis

#### Qualitative analysis

Braun and Clarke’s^38^ six-step thematic analysis, informed by the CFIR, will be used to understand the barriers and facilitators to implementation and potential solutions to the barriers. Two authors (AF and ML) will independently review each transcript and extract themes using a general inductive approach. A third author (MTB) will review the analyses and arbitrate discrepancies where necessary to reach consensus. Emerging themes will then be mapped to the CFIR domains and constructs. The researchers will meet periodically throughout the data analysis to discuss the themes, resolve discrepancies and reach a consensus on barriers, facilitators and proposed solutions. In Phase 2, qualitative findings will be integrated with implementation log data and quantitative implementation outcomes to address the process evaluation questions, with cross-cluster comparison used to explain variation in implementation and outcomes across diverse settings. Integration will follow a triangulation protocol, with qualitative themes, implementation log data and quantitative implementation outcomes brought together in joint display matrices by cluster, to identify areas of convergence, divergence and complementarity across data sources. The findings will inform the identification of barriers, facilitators and strategies to support the large-scale implementation and sustainability of ENABLE.

#### Quantitative analysis

All analyses will follow a predetermined statistical analysis plan. Data will be cleaned throughout the trial and at completion, with checks for outlying and implausible values. Participant characteristics and outcomes (overall and intervention versus control) will be summarised using descriptive statistics. Any substantive imbalances in prognostic variables will be adjusted for in the analyses.

#### Primary outcome

The primary outcome, the proportion of patients admitted to hospital with a chronic or complex wound, will be analysed using mixed-effects logistic regression, adjusted for important prognostic factors. P values, ORs and 95% CIs will be reported for treatment and control variables. Two levels of random effects will be included to account for clustering within sites and periods. The extent of data clustering will be assessed via determination of the variance partition coefficient of null models, and the model will be re-specified as necessary before the addition of covariates if the Variance Partition Coefficient (VPC) indicates very low levels of residual variance associated with a particular level. This model formulation is robust to missing data, but excessive amounts of missing data will be assessed, and any cases or variables with >50% missing data will be removed from the analysis. Intervention status will be modelled using a binary fixed effect. Secular trends will be accounted for with the inclusion of a fixed categorical effect. The analysis accounts for clustering within sites and repeated measurements within individuals, while adjusting for time period. We will allow secular trends to vary across sites via the inclusion of a random cluster-by-period affect. Model goodness-of-fit, linearity, calibration, classification and discrimination will be assessed, and residual analysis will be conducted to identify potentially influential data points.

#### Secondary outcomes

Length of hospital stay, HRQOL and wound percentage reduction will be modelled using mixed linear models, with p values, parameter estimates and 95% CIs reported for treatment and control variables. Data clustering for the secondary outcomes will be similar to that for the primary outcome.

Patient HRQOL will be analysed using the EQ-5D-5L, with improvement defined as a change from baseline (relative to control). Utility weights will be estimated from the EQ-5D-5L using Australian population-generated algorithms for EQ-5D-5L to calculate QALYs.^39^

#### Economic evaluation

A within-trial cost-effectiveness and cost-utility analysis comparing intervention and standard care groups will be undertaken from the perspective of the public healthcare funder.

Healthcare costs will be estimated by applying national weighted activity units and the national efficient price to activity-based data. Costs for non-hospital services will be derived from Medicare Benefits Schedule and Pharmaceutical Benefits Scheme datasets. Implementation costs (training, implementation support time, site onboarding and adaptation activities, drawn from the implementation logs) will be identified and reported separately from intervention delivery costs (specialist service time, app licensing and clinical consumables); research-related expenses will be excluded. Both cost categories will be included in the base-case analysis, with a sensitivity analysis excluding one-off implementation costs to reflect steady-state delivery. Analyses will account for variations in service delivery and funding models by estimating costs separately by clinic type.

Cost-effectiveness will be evaluated based on the incremental cost-effectiveness ratios for QALYs (cost-utility analysis) gained and cost per clinically meaningful improvement in EQ-5D-5L scores.

Additional analyses will be conducted to estimate the costs per hospital admission avoided for wound-related care. All intervention and healthcare resource costs over the trial duration will be included, with discounting applied at 5% per annum.

Uncertainty will be addressed using bootstrapping to generate confidence intervals around cost and outcome estimates. A cost-effectiveness acceptability curve will be plotted to show the probability that the intervention is cost-effective, given the differences in willingness-to-pay values for each additional health outcome achieved. Subgroup analyses for each clinic type will be undertaken. Aggregated cohort-level data will be summarised descriptively to compare service-level costs and outcomes before and after implementation across clusters and study periods.

## Ethics and dissemination

### Approval

This study has received ethical approval from the Sydney LHD HREC (Royal Prince Alfred Hospital Zone; protocol no.: X24-0360; ethics identifier: 2024/ETH02394) on 21 March 2025. The current protocol is V.7.0, approved 10 February 2026.

### Protocol amendments

All participating sites will be informed of any protocol amendments as soon as approval is received from the HREC. Approval for each amendment must also be obtained from the research governance office at each site before the amendment takes effect. The clinical trial was registered in the Australian New Zealand Clinical Trials Registry on 2 June 2025 (trial registration number ACTRN12625000562471).

### Consent and confidentiality

Informed written consent will be obtained from all participants by the research team. Participants will be provided with written information about the study and given adequate time to consider participation and ask questions before providing consent. Where applicable, additional consent will be sought for the collection and use of participant data for ancillary analyses. No biological specimens will be collected as part of this study.

Personal information will be collected and managed in accordance with applicable privacy legislation. Participant data will be de-identified and stored securely in password-protected systems, with access restricted to authorised study personnel. Data sharing will occur only in de-identified form. Confidentiality of all information will be maintained before, during and after the trial.

### Ancillary and post-trial care

Participants requiring ongoing care after phase 2 may continue to access the intervention for up to 12 months following site completion. Any injuries or complications potentially related to the intervention will be managed by the study team, including assistance in organising appropriate medical care. Eligible participants may receive free treatment as public patients in the Australian healthcare system.

Participants retain the right to seek compensation for any injury or complication resulting from the study and do not waive any legal rights by participating. The sponsor will adhere to Medicines Australia’s *Guidelines for Compensation for Injury Resulting from Participation in a Company-Sponsored Clinical Trial*.

### Dissemination and reporting

The study findings will be disseminated through peer-reviewed publications, conference presentations, stakeholder briefings and targeted educational activities with clinicians, health service leaders and policymakers to support translation, scaling and sustainability of the ENABLE model.

The findings will be reported in accordance with established reporting guidelines appropriate to each study component. Clinical effectiveness outcomes from the SW-CRT will be reported in line with the Consolidated Standards of Reporting Trials (CONSORT) SW-CRT extension.^40^ Implementation outcomes arising from the CFIR-guided^20^ mixed-methods evaluation will be reported according to the Standards for Reporting Implementation Studies^41^ and the Consolidated Criteria for Reporting Qualitative Research.^42^ Economic outcomes will be reported in accordance with the Consolidated Health Economic Evaluation Reporting Standards.^43^

### Expected outcomes

The ENABLE study commenced in September 2024 and will be completed by August 2027. The primary findings will be disseminated following completion of data collection and analysis, once the study has addressed its primary and secondary objectives. Given that ENABLE has been developed using an established implementation science framework and CFIR-guided^20^ design principles and informed by recent research on chronic wound care and integrated models of care, we anticipate that it will be feasible, acceptable and clinically meaningful across diverse healthcare settings.

This multidisciplinary and cross-sector collaboration will ensure that ENABLE will be grounded in lived experiences, clinically relevant and aligned with strategic digital health and health service priorities, strengthening its relevance, usability and scalability.

Through its pragmatic, multisite design, the ENABLE study is expected to generate robust real-world evidence on the effectiveness of an integrated model of wound care for people with chronic and complex wounds. We anticipate improvements in clinical outcomes, including wound healing trajectories, a reduction in wound-related complications, hospital re-admissions, and improved continuity of care. In addition, we expect improvements in health service outcomes, including more efficient care pathways, reduced unwarranted practice variations and better alignment of care with evidence-based guidelines.

Consistent with the implementation-focused nature of the study, we also expect to identify key barriers and facilitators influencing adoption, fidelity and sustainability of the ENABLE model across participating sites. These findings will be used to refine the intervention and implementation strategy, ensuring adaptability to local contexts while maintaining core model integrity. It is anticipated that clusters with higher baseline digital maturity and stronger interdisciplinary integration will demonstrate more rapid and sustained uptake of the ENABLE model. Sustainability findings will inform the translation consortium’s scale-up and sustainability planning with NSW Health partners.

NSW Health is currently adopting the Single Digital Patient Record (SDPR) technology for inpatient and outpatient care. We envisage that the SDPR will be able to support the ENABLE model of care in public hospital settings. If SDPR is eventually integrated into primary and residential aged care settings, we anticipate that it will also support the ENABLE model of care in these settings.

We acknowledge some methodological limitations of this SW-CRT.^40^ Although the randomisation of clusters will occur following site consent to reduce selection bias, concealing the initiation of the intervention will not be feasible given its system-level nature. Therefore, blinding of clinicians and participants is not possible; however, the use of objective outcome measures and routinely collected clinical and administrative data will minimise the risk of measurement bias. Recruitment bias is expected to be limited given the clearly defined eligibility criteria and pragmatic design embedded within usual care.

Despite these limitations, the SW-CRT design enhances internal validity while supporting strong external validity and generalisability across heterogeneous healthcare settings. The inclusion of multiple service types and care environments will ensure that the findings are applicable to real-world contexts where integrated wound care models either differ or are absent.

## Supplementary material

10.1136/bmjopen-2026-123987 online supplemental file 1

10.1136/bmjopen-2026-123987 online supplemental file 2

10.1136/bmjopen-2026-123987 online supplemental file 3

10.1136/bmjopen-2026-123987 online supplemental file 4

10.1136/bmjopen-2026-123987 online supplemental file 5

10.1136/bmjopen-2026-123987 online supplemental file 6

10.1136/bmjopen-2026-123987 online supplemental file 7

10.1136/bmjopen-2026-123987 online supplemental file 8

10.1136/bmjopen-2026-123987 online supplemental file 9

10.1136/bmjopen-2026-123987 online supplemental file 10

10.1136/bmjopen-2026-123987 online supplemental file 11

## Footnotes

Australia’s national regulatory authority responsible for evaluating, assessing and monitoring products defined as therapeutic goods.
