# Supplementary material for: Implementing a digitally enabled wound model of care to improve chronic wound outcomes in seven health services in Australia: study protocol for a multisite, hybrid type 2, two-phase effectiveness-implementation trial (the ENABLE study)
Source: BMJ Open. 2026 Nov 3;16(11):e123987. doi: 10.1136/bmjopen-2026-123987 (PMC13735325; doi:10.1136/bmjopen-2026-123987)
Supplement: Supplementary file 3 [file bmjopen-16-11-s003.pdf]

Participant Information Sheet/Consent Form

Interventional Study - *Adult providing own consent*

**Title**	*Improving patient outcomes and reducing the hidden burden of chronic wounds: a digitally enabled Wound Model of Care (The ENABLE Study)*
**Short Title**	ENABLE
**Protocol Number**	*X24-0360*
**Project Sponsor**	*University of Sydney*
**Coordinating Principal Investigator**	[redacted]
**Sites**	• Sydney Local Health District<br>• Western New South Wales Local Health District<br>• Murrumbidgee Local Health District<br>• South Western Sydney Local Health District<br>• Healthy North Coast<br>• Central Eastern Sydney Primary Health Network<br>• Wound Rescue
**Site Investigators**	• [redacted], Sydney Local Health District<br>• [redacted], Western New South Wales Local Health District<br>• [redacted], Murrumbidgee Local Health District<br>• [redacted], South Western Sydney Local Health District<br>• [redacted], Healthy North Coast<br>• [redacted], Central and Eastern Sydney Primary Healthcare Network<br>• [redacted], Wound Rescue

Part 1 What does my participation involve?

1 Introduction

You are invited to take part in this research project involving a clinical trial of an intervention for people with chronic or complex wounds. You are invited to take part in this clinical trial because you have a chronic wound or complex wound. The research project is testing a new model of care for chronic and complex wounds. It will involve accessing a specialist wound service (senior nurses who specialise in wound care) virtually and a wound app on your clinician's work phone that will allow your clinician and/or the specialist wound service to monitor your wound healing and the option of you having the wound app on your phone to take images of your wound in between your face-to-face appointments with your clinician.

This Participant Information Sheet/Consent Form tells you about the research project. It explains what is involved. Knowing what is involved will help you decide if you want to take part in the research.

Please read this information carefully. Ask questions about anything that you don't understand or want to know more about. Before deciding whether or not to take part, you might want to talk about it with a relative, friend or your local doctor.

Participation in this research is voluntary. If you don't wish to take part, you don't have to. You will receive the best possible care whether or not you take part.

If you decide you want to take part in the research project, you will be asked to sign the consent section. By signing it you are telling us that you:
- Understand what you have read
- Consent to take part in the research project
- Consent to have the tests and treatments that are described
- Consent to the use of your personal and health information as described.

You will be given a copy of this Participant Information and Consent Form to keep.

2 What is the purpose of this research?

The aim of this study is to implement a new model of care that uses the latest digital health innovations to enhance the way patients receive wound care. This model of care is the first of its kind in Australia to be trialled and will inform how it can be implemented in different settings in Australia.
This includes access to a specialist wound service, your clinician using a wound app to capture images of your wound and to monitor its progression and the option of you having access to the wound app on your phone (the app is called Tissue Analytics). The model of care is flexible, and you do not have to use the wound app on your phone if you don't want to.
Medications, drugs and devices have to be approved for use by the Australian Federal Government. Tissue Analytics is approved in Australia by the Therapeutic Goods Administration to treat chronic/complex wounds (ARTG ID 374066).
This research has been initiated by [redacted], Executive Research and Clinical Lead, Skin Integrity and Nurse Manager, University of Sydney and Sydney Local Health District. This research is being coordinated and conducted by The University of Sydney.

3 What does participation in this research involve?

You will be participating in a stepped wedge cluster randomised trial (a type of clinical trial) where you are in the intervention group and will receive the intervention if you provide your written consent. The consent form must be signed prior to any study assessments being performed or data being collected.
This research project has been designed to make sure the researchers interpret the results in a fair and appropriate way and avoids study personnel or participants jumping to conclusions.
There are no additional costs associated with participating in this research project, nor will you be paid. Access to the specialist wound service and the wound app, as part of the research project, will be provided to you free of charge.

To enrol in the study, you must firstly be established to have a chronic or complex wound (meet eligibility criteria), have read this Participant Information Sheet, asked the research officer any questions you may have, and have signed the consent form.

Once the consent form is signed you will be enrolled in the study by the research officer to receive the intervention (model of care). As part of the initial enrolment, you will complete a 5-question survey about your general health-related quality of life which will only take you 3 minutes to complete. You will have to complete this survey again on your final consultation for the wound (once it has healed), as well as another survey which has 5 demographic questions and 21 questions about your experience of the model of care. This survey will take 10 minutes to complete.

The intervention is flexible and will involve the clinician doing either one of the following:

- Use the digital wound app to take a wound image at every face-to-face wound consultation on their own work-issued phone AND engage in shared care with a specialist wound service
- Use the digital wound app to take a wound image at every face-to-face wound consultation on their own work-issued phone AND engage in shared care with the specialist wound service AND you/family member/carer will take a wound image at intervals in between face-to-face appointments according to the treating clinician's advice.

You can download the wound app on your own phone to capture images of your wound in between face-to-face appointments and your clinician will be able to remotely monitor your wound and communicate with you via the app and send you reminders to change the dressing (if required).

- Your treating clinician or research officer will guide you on how to download and use the app. This process will only take 10 minutes.

You will take ownership of the images taken on the app on your device, however, family members, carers, or other clinicians may be able to take the image for you for better image capture (e.g. they may have a wound in a position that may make it difficult to properly capture an image, such as their posterior body).
You can use the app to capture an image of your wound at intervals determined by your clinician who can set the reminders for you. You will receive a notification on your phone from their app when it is time to capture a new wound image. It will be up to your treating clinician to determine the frequency of the reminders, or if any reminders are required.

The clinician will consult a specialist wound service (senior nurses with expertise in wound care) who will engage in shared care with the specialist to manage the wound and provide expert advice.

The app will collect only wound-related data such as the image of the wound, type of wound, location, presence of infection, odour, exudate (secretions from the wound), and the wound dressing used. It is up to your discretion and your treating clinician to determine if you would like to download the app on your phone to monitor the wound.

Tissue Analytics transports all protected health information through the platform safely and securely. No patient information is ever stored on the mobile phone. Tissue Analytics uses industry best practices of encryption, and data collected through their system which is encrypted from end-to-end. Additionally, all data collected by Tissue Analytics is removed from the device immediately after being sent to the website for review. Their cloud, which is built using dedicated hardware on Amazon Web Services, so that all patient data that is entered is guaranteed to be safe and secure.

The study will require access to your wound and demographic data on Tissue Analytics, your medical record, and Medicare Benefits Schedule (MBS) and Pharmaceutical Benefits Scheme (PBS) data from Services Australia. You will be asked to sign a separate consent form authorising the study to access your Commonwealth health information provided by Services Australia, see the separate Services Australia Participant Information Document and Participant Consent Form. Services Australia is not involved in this research other than to provide the information that you have consented to the release of, should you decide to participate in this study. Services Australia has confirmed that this research and any associated documents have received approval from a Human Research Ethics Committee (HREC) that is registered with and operates within guidelines set out by the National Health and Medical Research Council (NHMRC).

You will remain enrolled in the study to receive the intervention until your wound has healed, or you wish to cease receiving the intervention, or your treating clinician determines that you no longer require the intervention.

The research at your site will be monitored by the research officer and the site investigator.

They will regularly report back to the Program Manager and Co-Ordinating Principal Investigator on the progress of the research and there will be governance structures (e.g. Committees) in place to monitor research activity at each site.

4 What do I have to do?

If you would like to download the wound app on your phone, please ensure your phone has the following:

- Camera
- Flash (If your device does not have a flash, ensure you have strong lighting on the wound.)
- Internet connectivity with minimum speeds of 4Mbps downstream and 1Mbps upstream.

The following mobile phones are known to work with Tissue Analytics:

- iPhone (SE, X or 10 and above)
- Pixel A Series (4a, 5a, etc)
- Samsung S Series, S20 or higher

If you have a mobile phone that is not in the list, you can still try to use it but may encounter difficulties with some features or functionalities.

The Tissue Analytics app will run on a tablet, but tablets come with two inherent disadvantages:

- Awkward Size: Tablets can be difficult to position for capturing photos of a wound because of their size and weight.
- No flash: Most tablets do not have a built-in flash, making it difficult to sufficiently illuminate the wound.

If you choose to download the wound app and want to take a picture of the wound on a part of your body that may make it difficult to capture, you can ask a family member or carer to take a clear picture for you on the app (the image if captured on the app, not on your phone's native camera).

Your treating clinician will be able to trigger notifications in your app to remind you to take a picture of your wound and can be set at regular intervals as clinically required.

5 Other relevant information about the research project

There are seven sites conducting this clinical trial with their patients:

- Sydney Local Health District
- Western New South Wales Local Health District
- Murrumbidgee Local Health District
- South Western Sydney Local Health District
- Healthy North Coast
- Central Eastern Sydney Primary Health Network
- Wound Rescue

This clinical trial aims to recruit 375 patients in the control group (will receive standard care) and 375 patients in the intervention group (will receive model of care). The project involves researchers from the above organisations as well as researchers from the University of Sydney (Executive Sponsor of the study), University of Queensland, and the University of Huddersfield. We are collaborating with Wounds Australia, NSW Agency for Clinical Innovation, and eHealth NSW on this project.

6 Do I have to take part in this research project?

Participation in any research project is voluntary. If you do not wish to take part, you do not have to. If you decide to take part and later change your mind, you are free to withdraw from the project at any stage.

If you do decide to take part, you will be given this Participant Information and Consent Form to sign and you will be given a copy to keep.

Your decision whether to take part or not to take part, or to take part and then withdraw, will not affect your routine treatment, your relationship with those treating you or your relationship with *[Institution]*.

7 What are the alternatives to participation?

You do not have to take part in this research project to receive the intervention at this service. Other options are available; these include standard care (the care you would have received if this study did not exist). Your treating clinician will discuss these options with you before you decide whether or not to take part in this research project.

8 What are the possible benefits of taking part?

We cannot guarantee that you will receive any benefits from this research; however, possible benefits may include benefits to future patients with chronic or complex wounds, enhancing their experience of wound care.

9 What are the possible risks and disadvantages of taking part?

We anticipate minimal risks or disadvantages to participating in this study.

There may be side effects that the researchers do not expect or do not know about and that may be serious. Tell your treating clinician immediately about any new or unusual symptoms that you get.

Many side effects go away shortly after treatment ends. However, sometimes side effects can be serious, long lasting or permanent. If a severe side effect or reaction occurs, your treating clinician may need to stop you receiving the intervention. Your treating clinician will discuss the best way of managing any side effects with you.

10 What if new information arises during this research project?

Sometimes during the course of a research project, new information becomes available about the treatment that is being studied. If this happens, your treating clinician will tell you about it and discuss with you whether you want to continue in the research project. If you decide to withdraw, your treating clinician will make arrangements for your regular wound care to continue. If you decide to continue in the research project you will be asked to sign an updated consent form.

Also, on receiving new information, your treating clinician might consider it to be in your best interests to withdraw you from the research project. If this happens, he/ she will explain the reasons and arrange for your regular wound care to continue.

11 Can I have other treatments during this research project?

This intervention will not have any implications for any other medications or treatments you take during this research project.

12 What if I withdraw from this research project?

If you decide to withdraw from the project, please notify the research officer before you withdraw. A Clinical Trial Withdrawal Form will be provided to you to sign. There are no health risks or special requirements linked to withdrawing.

If you do withdraw your consent during the research project, on the Clinical Trial Withdrawal Form you can choose to either withdraw from the study and request all your study data collected to be securely deleted or you can choose to withdraw from the study and allow the researchers to retain your study data for the purposes of the current research.

13 Could this research project be stopped unexpectedly?

This research project may be stopped unexpectedly for a variety of reasons, however, this is very unlikely. These may include reasons such as:

- Unacceptable side effects
- The intervention being shown not to be effective.

14 What happens when the research project ends?

At the end of the research project, you will continue to receive the intervention until your wound is healed, you withdraw from the study (in which case you will then receive standard care, at no extra cost to you), or your treating clinician advises you to cease the intervention. You will be emailed a summary of the study findings at the conclusion of the project if you provide your email address in the Consent Form.

Part 2 How is the research project being conducted?

15 What will happen to information about me?

By signing the consent form, you consent to the treating clinician and relevant research staff collecting and using personal information about you for the research project. Information about you may be obtained from your health records held at this and other health services for the purpose of this research. By signing the consent form you agree to the study team accessing health records if they are relevant to your participation in this research project.
Any information obtained in connection with this research project that can identify you will remain confidential. You will be assigned a participant study number and your identifying information will be stored separately from de-identified data. Only your participant study number will be attached to de-identified data. However, you will be re-identifiable in the event you decide to withdraw and request your data to be deleted.

The data will be stored electronically in a database named REDCap (Research Electronic Data Capture). This is a secure, web-based, non-commercial, data management tool designed for research purposes, hosted and backed up on the University of Sydney servers. Only the site research officer, site investigator, Co-ordinating Principal Investigator and Program Manager will have access to your data. Your information will only be used for the purpose of this research project and it will only be disclosed with your permission, except as required by law. Your data will be be used for future use and any data used or shared will be non-identifiable data with collaborators only for the purposes of research that are extensions of, or closely related to, the original project or in the same general area of research. By signing the consent form, you agree to this future use. All research data (electronic and hardcopy) will be retained for 15 years from the date of publication after which secure destruction of the research data will occur. The research project will not establish a databank. Your Services Australia information will not be used for unrelated research outside of the approved study.

Your health records and any information obtained during the research project are subject to inspection (for the purpose of verifying the procedures and the data) by the relevant authorities and authorised representatives of the Sponsor, the University of Sydney, the institution relevant to this Participant Information Sheet, [Name of institution], or as required by law. By signing the Consent Form, you authorise release of, or access to, this confidential information to the relevant study personnel and regulatory authorities as noted above.

It is anticipated that the results of this research project will be published and/or presented in a variety of forums. In any publication and/or presentation, information will be provided in such a way that you cannot be identified, including ensuring any individual identifying information is not presented and aggregated (summarised) data is presented, where appropriate.

Information about your participation in this research project may be recorded in your health records.

In accordance with relevant Australian and New South Wales privacy and other relevant laws, you have the right to request access to your information collected and stored by the research team. You also have the right to request that any information with which you disagree be corrected. Please contact the study team member named at the end of this document if you would like to access your information. Any information obtained for the purpose of this research project and for the future research described in Section 15 that can identify you will be treated as confidential and securely stored. It will be disclosed only with your permission, or as required by law.

16 Complaints and compensation

If you suffer any injuries or complications as a result of this research project, you should contact the study team as soon as possible and you will be assisted with arranging appropriate medical treatment. If you are eligible for Medicare, you can receive any medical treatment required to treat the injury or complication, free of charge, as a public patient in any Australian public hospital. In addition, you may have a right to take legal action to obtain compensation for any injuries or complications resulting from the study. Compensation may be available if your injury or complication is sufficiently serious and is caused by unsafe drugs or equipment, or by the negligence of one of the parties involved in the study (for example, the researcher, the hospital, or the treating doctor). You do not give up any legal rights to compensation by participating in this study.

17 Who is organising and funding the research?

This research project is being conducted by [redacted] and funded by a National Health and Medical Research Council (NHMRC) Partnership Project Grant.
You will not benefit financially from your involvement in this research project and no member of the research team will receive a personal financial benefit from your involvement in this research project (other than their ordinary wages).

NetHealth (manufacturer of Tissue Analytics) will provide support on establishing the Tissue Analytics platform at each site. There will be no transfer of any information to the manufacturer by the investigators. The manufacturer will be paid a subsidised fee for the investigators to use the platform for the purpose of this research, otherwise, no other financial benefit will be gained by the manufacturer. Only the investigators will have ownership over the study data, not the manufacturer.

18 Who has reviewed the research project?

All research in Australia involving humans is reviewed by an independent group of people called a Human Research Ethics Committee (HREC). The ethical aspects of this research project have been approved by the HREC of *Sydney Local Health District (RPAH Zone)*.

Approval has been given by the institution where this research is being carried out.<br><br>This project will be carried out according to the *National Statement on Ethical Conduct in Human Research (2023)*. This statement has been developed to protect the interests of people who agree to participate in human research studies.

19 Further information and who to contact

The person you may need to contact will depend on the nature of your query.<br><br>If you want any further information concerning this project or if you have any medical problems which may be related to your involvement in the project (for example, any side effects), you can contact your treating clinician or any of the following people:

Clinical contact person

Name	*[Name]*
Position	*[Position]*
Telephone	*[Phone number]*
Email	*[Email address]*

For matters relating to research at the site at which you are participating, the details of the local site complaints person are:

Complaints contact person

Name	*[Name of Site Investigator]*
Position	*[Position]*
Telephone	*[Phone number]*
Email	*[Email address]*

If you have any complaints about any aspect of the project, the way it is being conducted or any questions about being a research participant in general, then you may contact:

Reviewing HREC approving this research and HREC Executive Officer details

Reviewing HREC name	*Sydney Local Health District Ethics Review Committee (RPAH Zone)*
HREC Executive Officer	HREC Executive Officer
Telephone	[redacted]
Email	[redacted]
Protocol Number	*[Protocol Number X24-0360]*

Local HREC Office contact (Single Site - Research Governance Officer)

Name	*[Name]*
Position	*[Position]*
Telephone	*[Phone number]*
Email	*[Email address]*

Consent Form - *Adult providing own consent*

**Title**	*Improving patient outcomes and reducing the hidden burden of chronic wounds: a digitally enabled Wound Model of Care (The ENABLE Study)*
**Short Title**	*The ENABLE Study*
**Protocol Number**	*X24-0360*
**Project Sponsor**	*University of Sydney*
**Coordinating Principal Investigator**	[redacted] *Executive Research and Clinical Lead of Skin Integrity, Sydney Local Health District and University of Sydney*
**Associate Investigator(s)**	*[Associate Investigator(s) of Site]*
**Location**	*[Site]*

Declaration by Participant

I have read the Participant Information Sheet or someone has read it to me in a language that I understand.

I understand the purposes, procedures and risks of the research described in the project.

I give permission for my treating clinicians outside this service to release information (including data concerning me and my wound from the medical records and digital wound platform) to the University of Sydney concerning my disease and treatment for the purposes of this project. I understand that such information will remain confidential.

I understand that my participation in this study will allow the researchers and others, as described in the Information for Participants, to have access to my medical record, and I agree to this.

I give permission for my data to be used for future use and any data used or shared will be non-identifiable data with collaborators only for the purposes of research that are extensions of, or closely related to, the original project or in the same general area of research.

I have had an opportunity to ask questions, and I am satisfied with the answers I have received.

I freely agree to participate in this research project as described and understand that I am free to withdraw at any time during the study without affecting my future health care.

I understand that I will be given a signed copy of this document to keep.

Name of Participant (please print) ______________________________

Signature ______________________ Date ______________________

I would like to receive a summary of the study findings at study completion to the email below:

Email address: ______________________________

Declaration by Study Doctor/Senior Researcher†

I have given a verbal explanation of the research project, its procedures and risks and I believe that the participant has understood that explanation.

Name of Study Doctor/
Senior Researcher† (please print) ______________________________

Signature ______________________ Date ______________________

† A senior member of the research team must provide the explanation of, and information concerning, the research project.

Note: All parties signing the consent section must date their own signature.
