# Supplementary material for: Implementing a digitally enabled wound model of care to improve chronic wound outcomes in seven health services in Australia: study protocol for a multisite, hybrid type 2, two-phase effectiveness-implementation trial (the ENABLE study)
Source: BMJ Open. 2026 Nov 3;16(11):e123987. doi: 10.1136/bmjopen-2026-123987 (PMC13735325; doi:10.1136/bmjopen-2026-123987)
Supplement: Supplementary file 5 [file bmjopen-16-11-s005.pdf]

[Site logo]

Improving patient outcomes and reducing the hidden burden of chronic wounds: a digitally enabled Wound Model of Care (The ENABLE Study)

PARTICIPANT INFORMATION SHEET – CLINICIAN INTERVIEWS/FOCUS GROUPS (POST-IMPLEMENTATION)

**Short Title**	ENABLE
**Project Sponsor**	University of Sydney
**Coordinating Principal Investigator**	[redacted]
**Site Investigator**	[Name of Site Investigator]
**Site**	[SITE]

1. Introduction

You are invited to take part in a research study into people's experiences of having a wound, the wound care they received, and your recent experience of the digital wound model of care implemented in [SITE]. The model of care involved access to a specialist wound service and a digital wound platform. We will be exploring how it was implemented, what went well and what could be improved on. Your thoughts are very important to us to make sure we are meeting the needs of patients with chronic and complex wounds and ensure we can provide the best care for them.

You have been invited to take part in this study as you accessed the new digital wound model of care at [SITE] for your patients with chronic or complex wounds.

This study is part of a wider project that implemented at [SITE] a new digital wound model of care involving access to a specialist wound service and a wound app.

The study is being conducted at the following institution and led by the following Site Investigators:

Site	Site Investigator	Position
Sydney Local Health District	[redacted]	Clinical Nurse Consultant, Skin Integrity, Digital Wound Care, and Wound Command Centre
Western New South Wales Local Health District	[redacted]	Clinical Nurse Consultant, Wound Prevention and Management
Murrumbidgee Local Health District	[redacted]	Nurse Manager, Comprehensive Care
South Western Sydney Local Health District	[redacted]	Director of Strategy and Partnerships
Healthy North Coast	[redacted]	Primary Health Coordinator
Central and Eastern Sydney Primary Healthcare Network	[redacted]	General Manager, Primary Care Improvement
Wound Rescue	[redacted]	Director

The study is being sponsored by the University of Sydney and is supported by a research grant from the National Health and Medical Research Council (NHMRC) with a Partnership Project Grant.

There is also a student-investigator on this study, [redacted]. This interview will be conducted by [redacted] who is collecting data in the study to partially fulfill the requirements of a Master of Philosophy degree at the University of Sydney under the supervision of under the supervision of [redacted] (Co-Ordinating Principal Investigator) and [redacted] (Chief Investigator). Analysis will be conducted at the University of Sydney.

This Participant Information Sheet (PIS) will tell you what is involved in the study and help you decide whether or not you wish to take part. Please read this information carefully. If there is anything you do not understand or if you feel you need more information about anything, please ask.
Before you make a decision, please feel free to talk things over with your line manager.

2. Study Procedures

If you agree to participate in this study, you will be asked to sign the Participant Consent Form at the end of this document by the site research officer. You will then be asked to undergo the following study activities:

- Complete a brief demographic questionnaire with 4 questions on an online REDCap survey – the research officer will provide you with the survey link.
- Participate in an interview that will last approximately 30-45 minutes. The interview will be conducted by [redacted]. You will be asked ten questions about your experience exploring how the new digital wound model of care was implemented at [SITE], what went well and what could be improved on.
- The interview will be conducted either in-person at [SITE], virtually (via videoconference on Teams or Zoom) or via phone at a time convenient for you.
- The Interview will be audio-recorded.
- [redacted] will take notes during and/or immediately after the interview.

If you agree to participate in this study, you will be asked to sign the Participant Consent Form prior to participating in a (maximum 45 minutes) audio-recorded interview. This audio-recording will then be transcribed using Microsoft Word and reviewed by the site research officer and will not contain any details that will identify you.

3. Risks

There are no foreseeable risks associated with this study.

4. Benefits

While we intend that this research study furthers medical knowledge and may improve treatment of chronic or complex wounds in the future, it may not be of direct benefit to you.

5. Costs

Participation in this study will not cost you anything, nor will you be paid.

6. Voluntary Participation

Participation in this study is entirely voluntary. You do not have to take part in it. If you do take part, you can withdraw at any time without having to give a reason by contacting

[researcher name and contact number and email]. Whatever your decision, please be assured that it will not affect your relationship with the research team.

If you decide to withdraw from the study, we will not collect any more study-related information from you.

If you want to withdraw, please let us know and tell us what you would like us to do with the information we have collected from you up till then. If you wish, your information will be removed from our study records. It will not be included in the study results, unless we have analysed and published the results.

Data collected up until the time you withdraw may be included in the study. If you do not want the researchers to do this, you must tell them at the time of your withdrawal. The study results may be presented at a conference or in a scientific publication, but individual participants will not be identifiable in such a presentation.

7. Confidentiality

All the information collected from you for the study will be treated confidentially and will be stored on a research database in the University of Sydney’s Highly Protected SharePoint. Only the site research officer, site investigator, Co-Ordinating Principal Investigator and Program Manager will have access to it.

The data will be analysed by the researchers at the University of Sydney. All data for use in journal publications and presentations will be de-identified (de-identified data means that you/your information will not be identifiable). The files will be retained for 5 years from the day the study is completed. Once the retention expires the files will be disposed of via secure deletion.

Identified data will be stored on the University of Sydney’s Highly Protected SharePoint, an enterprise content management tool developed by Microsoft that meet the University's security requirements for storing and accessing Highly Protected data and facilitating secure collaboration with external collaborators. It is supported by the *University of Sydney’s Information Technology Department.* Your signed consent and the interviewer’s field notes will be stored in a locked cabinet in a locked office at [SITE].

8. Storage of Data

The University of Sydney software licence for REDCap (Research Electronic Data Capture) will be used for to manage the collection and storage of research data. REDCap is a secure, web-based, non-commercial, data management tool designed for research purposes. Data collected by REDCap is stored on servers in the University of Sydney data centre. Data is secured and regularly backed-up to protect privacy and confidentiality.

9. Future use of Data

With your permission, the data collected in this project may also be used in future research studies. The results of this study and non-identified raw data may also be shared in the future with national and international collaborators. If any stored data are used for future research, the research will first be reviewed and approved by an appropriately constituted Ethics Committee.

You can indicate your agreement to this on the Participant Consent Form.

10. Further Information

When you have read this information, [name of site research officer] will discuss it with you further and answer any questions you may have. If you would like to know more at any stage, please feel free to contact the site investigator, [name of site investigator] on [telephone number] or [email address].

This information sheet is for you to keep.

11. Ethics Approval and Complaints

This study has been approved by the Ethics Review Committee (RPAH Zone) of the Sydney Local Health District. Any person with concerns or complaints about the conduct of this study should contact the Executive Officer on [redacted] or [redacted] and quote protocol number X24-0360.
