# Supplementary material for: Implementing a digitally enabled wound model of care to improve chronic wound outcomes in seven health services in Australia: study protocol for a multisite, hybrid type 2, two-phase effectiveness-implementation trial (the ENABLE study)
Source: BMJ Open. 2026 Nov 3;16(11):e123987. doi: 10.1136/bmjopen-2026-123987 (PMC13735325; doi:10.1136/bmjopen-2026-123987)
Supplement: Supplementary file 6 [file bmjopen-16-11-s006.pdf]

[SITE LOGO]

Patient experience of Wound Care

Please complete the following survey on your experience of wound care. This survey will take approximately 10 minutes to complete.

Patient Experience of Wound Care

**Date survey completed**	
**Who completed this survey?**	☐ Patient<br>☐ Someone else on behalf of the patient
**Age**	☐ 18 to 24 years<br>☐ 25 to 34 years<br>☐ 35 to 44 years<br>☐ 45 to 54 years<br>☐ 55 to 64 years<br>☐ 65 to 74 years<br>☐ 75 years or older
**Gender**	☐ Male<br>☐ Female<br>☐ Other
**Language spoken at home**	☐ English<br>☐ Other, please specify:
**Do you need an interpreter for your care?**	☐ Yes<br>☐ No

Please answer the questions below by selecting the response that best reflects your experience, using the following ratings. If you are a carer, please answer on behalf of the patient and their experience.
0= Strongly Disagree/ 1= Disagree/ 2=Neutral/ 3=Agree/ 4=strongly agree

	**Strongly Disagree**	**Disagree**	**Neutral**	**Agree**	**Strongly agree**
**1. The current wound care provided to me is effective**	0	1	2	3	4
**2. I know who to contact if I have concerns about my wound care**	0	1	2	3	4
**3. It is easy for me to contact my wound care provider if I need further help with my wound**	0	1	2	3	4

	Strongly Disagree	Disagree	Neutral	Agree	Strongly agree
**4. If I raise a concern about my wound care, my wound care provider responds promptly**	0	1	2	3	4
**5. I find the need to travel for wound care a burden (for example financially, loss of time, physically tiring)**	0	1	2	3	4
**6. I was confident in the care I received from the wound care provider**	0	1	2	3	4
**7. I was well informed about my wound care plan**	0	1	2	3	4
**8. I was confident to follow the wound care provider's instructions on how to look after my wound**	0	1	2	3	4
**9. My wound care provider made it easy for me to get treatment**	0	1	2	3	4
**10. I was involved as much as I wanted in making decisions about my condition and care needs**	0	1	2	3	4
**11. My family and/or carer were involved as much as I wanted in making decisions about my condition and care needs**	0	1	2	3	4
**12. I would recommend my wounder care provider to others**	0	1	2	3	4
**13. Overall, how would you rate the care you received from the wound care provider?**	0 Very poor	1 Poor	2 Neutral	3 Good	4 Excellent

Thank you for completing this survey.
