# Supplementary material for: Implementing a digitally enabled wound model of care to improve chronic wound outcomes in seven health services in Australia: study protocol for a multisite, hybrid type 2, two-phase effectiveness-implementation trial (the ENABLE study)
Source: BMJ Open. 2026 Nov 3;16(11):e123987. doi: 10.1136/bmjopen-2026-123987 (PMC13735325; doi:10.1136/bmjopen-2026-123987)
Supplement: Supplementary file 7 [file bmjopen-16-11-s007.pdf]

[SITE LOGO]

Patient experience of Digital Wound Model of Care

Please complete the following survey on your experience of the Digital Wound Model of Care (specialist wound service and digital wound app [Tissue Analytics]). This survey will take approximately 10 minutes to complete.

Patient Experience of Specialist Wound Service

**Date survey completed**	
**Who completed this survey?**	☐ Patient<br>☐ Someone else on behalf of the patient
**Age**	☐ 18 to 24 years<br>☐ 25 to 34 years<br>☐ 35 to 44 years<br>☐ 45 to 54 years<br>☐ 55 to 64 years<br>☐ 65 to 74 years<br>☐ 75 years or older
**Gender**	☐ Male<br>☐ Female<br>☐ Other
**Language spoken at home**	☐ English<br>☐ Other, please specify:
**Do you need an interpreter for your care?**	☐ Yes<br>☐ No

Please answer the questions below by selecting the response that best reflects your experience, using the following ratings. If you are a carer, please answer on behalf of the patient and their experience.
0= Strongly Disagree/ 1= Disagree/ 2=Neutral/ 3=Agree/ 4=strongly agree

	Strongly Disagree	Disagree	Neutral	Agree	Strongly agree
**1. The specialist wound service provided me with an effective wound care service**	0	1	2	3	4
**2. I know who to contact if I have concerns about my wound care**	0	1	2	3	4

**3. It is easy for me to contact my wound care provider if I need further help with my wound**	0	1	2	3	4
**4. How did virtual care benefit you?**<br><br>(Virtual care includes the Digital Wound app, video and phone appointments and any other electronic communication with your wound care provider)	Tick all that applies.<br>☐ I was able to reduce my need to travel<br>☐ I saved money<br>☐ I did not miss out on work or school<br>☐ I could involve my family in my care<br>☐ I did not need to arrange care for my child and/or dependent/s<br>☐ It helped me understand and manage my condition<br>☐ It benefited me in other ways, please specify below:				
	**Strongly Disagree**	**Disagree**	**Neutral**	**Agree**	**Strongly agree**
**5. If I raise a concern about my wound care, my wound care provider responds promptly**	0	1	2	3	4
**6. I find the need to travel for wound care a burden (for example financially, loss of time, physically tiring)**	0	1	2	3	4
**7. I was confident in the care I received from the wound care provider**	0	1	2	3	4
**8. I was well informed about my wound care plan**	0	1	2	3	4
**9. I was confident to follow the wound care provider's instructions on how to look after my wound**	0	1	2	3	4
**10. The specialist wound service made it easy for me to get treatment**	0	1	2	3	4
**11. I was involved as much as I wanted in making decisions about my condition and care needs**	0	1	2	3	4
**12. My family and/or carer were involved as much as I wanted in making decisions about my condition and care needs**	0	1	2	3	4
**13. I would recommend the specialist wound service to others**	0	1	2	3	4

**14. Overall, how would you rate the care you received from the specialist wound service?**	0<br>Very poor	1<br>Poor	2<br>Neutral	3<br>Good	4<br>Excellent

Patient experience of Tissue Analytics Digital Wound App

**15. Did you use the Tissue Analytics digital wound app?**	☐ Yes<br>☐ No (End of survey)
**16. The Tissue Analytics digital wound app was used by… (select all that apply)**	☐ Myself<br>☐ Family member<br>☐ Friend<br>☐ Clinician
**17. I used the Tissue Analytics digital wound app to monitor my wound progress**	☐ Never<br>☐ Rarely<br>☐ Sometimes<br>☐ Often<br>☐ Always

**Please answer the questions below by selecting the response that best reflects your experience, using the following ratings<br>0= Strongly Disagree/ 1= Disagree/ 2=Neutral/ 3=Agree/ 4=strongly agree**					
	**Strongly Disagree**	**Disagree**	**Neutral**	**Agree**	**Strongly agree**
**18. It was easy for me (or other users) to take images of my wound using the Tissue Analytics digital wound app**	0	1	2	3	4
**19. The Tissue Analytics digital wound app requires internet access and this was not a problem for me**	0	1	2	3	4
**20. The Tissue Analytics digital wound app increased my confidence to look after my wound**	0	1	2	3	4
**21. The Tissue Analytics digital wound app improved communication between me and the wound care provider**	0	1	2	3	4

Thank you for completing this survey.
