# Supplementary material for: Implementing a digitally enabled wound model of care to improve chronic wound outcomes in seven health services in Australia: study protocol for a multisite, hybrid type 2, two-phase effectiveness-implementation trial (the ENABLE study)
Source: BMJ Open. 2026 Nov 3;16(11):e123987. doi: 10.1136/bmjopen-2026-123987 (PMC13735325; doi:10.1136/bmjopen-2026-123987)
Supplement: Supplementary file 8 [file bmjopen-16-11-s008.pdf]

Patient Interview Guide – Pre-Implementation of Digital Wound Model of Care

Introduction

Thank you for consenting to participate in this interview. My name is [name of interviewer] and I'm a [role of interviewer]. We are running this research to understand people's experiences of having a wound, the wound care they received, and their thoughts on an upcoming new way to receive wound care. Your experience is very important to us to make sure we are meeting patient needs and ensure we can provide the best care we can for future patients. We plan to analyse and publish the results from these interviews.

- *Confirm the participant has read the Participant Information Sheet and answer any queries which arise*
- *Confirm they are still willing to participate*
- *Confirm the participant is happy for the interview to be audio-recorded*

I am recording today's interview for transcription but as stated in the Participant Information Sheet, you will not be identified in any publications and if you do mention anything that might identify you such, it will be *censored. Once the interview has been transcribed the recording will be deleted.*

We can take a break or stop at any time, you do not need to give me a reason for stopping and it will not impact on your relationship with any staff at [organisation].

- *Are you okay with everything I just mentioned?*
- *Do you have any questions?*
- *Happy to proceed?*

Demographics (to be completed by interviewer)

Gender

☐ Male ☐ Female ☐ Other

Number of Wounds ____

Wound Aetiology/Type____

Wound Location ____

Wound Duration ____

Questions

1. Can you describe what it's like living with a wound and how it has impacted your daily life?
    a. Prompt: Explore physical, emotional and social impacts or in other aspects of life as a result of the wound
2. Could you share details about the treatment you received for your wound? What did you think of the treatment?

   a. Prompt: Treatment includes the management and care of the wound, clinic attending etc.
3. What impact did the treatment have on your health and well-being?
4. There is a new way of receiving wound care where, in between your appointments with your clinician, your wound care is managed virtually by them through an app on your phone or your carer's phone, and you or your carer will use the app to send pictures of your wound to the clinician.
   a. Prompt: Do you think this is something you or your carer could do? Why? Why not?
   b. Prompt: Can you describe how comfortable or confident you would feel using an app to monitor your wound?
   c. Prompt: Any concerns about using an app to monitor wound? What are they?
5. How would you feel about your clinician consulting wound care specialists outside of [organisation] for additional expert advice on your treatment? *(Only applicable to sites that will access Wound Care Command Centre at RPA Virtual Hospital, Sydney Local Health District)*.
6. Is there anything else you'd like to share about your wound or your overall experience with the care you've received?
