# Supplementary material for: Implementing a digitally enabled wound model of care to improve chronic wound outcomes in seven health services in Australia: study protocol for a multisite, hybrid type 2, two-phase effectiveness-implementation trial (the ENABLE study)
Source: BMJ Open. 2026 Nov 3;16(11):e123987. doi: 10.1136/bmjopen-2026-123987 (PMC13735325; doi:10.1136/bmjopen-2026-123987)
Supplement: Supplementary file 9 [file bmjopen-16-11-s009.pdf]

Patient Interview Guide – Post-Implementation of Digital Wound Model of Care

<u>*Introduction*</u>
Thank you for consenting to participate in this interview. My name is [name of interviewer] and I'm a [role of interviewer]. I am conducting research into the experiences of having a wound, the wound care you received, and your experience of this new way to receive wound care. Your experience is very important to us to make sure we are meeting future patient needs and ensure we can provide the best care we can for future patients. We plan to analyse and publish the results from these interviews.
• Confirm the participant has read the Participant Information Sheet and answer any queries which arise
• Confirm they are still willing to participate
• Confirm the participant is happy for the interview to be audio-recorded
I am recording today's interview for transcription but as stated in the Participant Information Sheet, you will not be identified in any publications and if you do mention anything that might identify you such, it will be *censored. Once the interview has been transcribed the recording will be deleted.*
We can take a break or stop at any time, you do not need to give me a reason for stopping and it will not impact on your relationship with any staff at [organisation].
• Are you okay with everything I just mentioned?
• Do you have any questions?
• Happy to proceed?

<u>**Demographics**</u> (to be completed by interviewer)

Gender

☐ Male ☐ Female ☐ Other

Number of Wounds ____

Wound Aetiology/Type____

Wound Location ____

Wound Duration ____

Questions

1. Can you describe what it's like living with a wound and how it has impacted your daily life?
    a. Prompt: Explore physical, emotional and social impacts or in other aspects of life as a result of the wound
2. Could you share details about the treatment you received for your wound? What did you think of the treatment?
    a. Prompt: Treatment includes the management and care of the wound, clinic attending etc.

3. What did you think of the approach to wound care you experienced? And how did it compare to traditional face-to-face consultations?
    a. Prompt: Different communication, support, outcomes?
4. Can you describe how you, your carer, or your clinician used the app, if at all, to take pictures of your wound during or between appointments?
    a. Prompt: (If used) Can you describe the process of using the app?
    b. Prompt: (If used) Was the app easy to learn and to use? Would you use it again?
5. Did your clinician consult wound experts outside of [organisation] for specialist wound advice? *(Only applicable to sites that will access Wound Care Command Centre at RPA Virtual Hospital, Sydney Local Health District)*.
    a. Prompt: (If yes) Can you tell me about what it involved?
6. How has this approach to wound care influenced your overall well-being?
7. What additional thoughts or experiences would you like to share about your wound and the care you've received?
