# Supplementary material for: Implementing a digitally enabled wound model of care to improve chronic wound outcomes in seven health services in Australia: study protocol for a multisite, hybrid type 2, two-phase effectiveness-implementation trial (the ENABLE study)
Source: BMJ Open. 2026 Nov 3;16(11):e123987. doi: 10.1136/bmjopen-2026-123987 (PMC13735325; doi:10.1136/bmjopen-2026-123987)
Supplement: Supplementary file 10 [file bmjopen-16-11-s010.pdf]

Clinician Interview Guide – Pre-Implementation of Digital Wound Model of Care

<u>*Introduction*</u>
Thank you for consenting to participate in this interview/focus group. My name is [name of interviewer] and I'm a [role of interviewer]. I am conducting research to understand clinicians' experiences of providing wound care and their thoughts on how a new model of wound care could be implemented in organisations. The model of care involves access to a specialist wound service and a digital wound platform. Your thoughts are very important to us to make sure we are meeting the needs of patients with chronic and complex wounds and ensure we can provide the best care for them. We plan to analyse and publish the results from these interviews.
- *Confirm the participant has read the Participant Information Sheet and answer any queries which arise*
- *Confirm they are still willing to participate*
- *Confirm the participant is happy for the interview to be audio-recorded*

I am recording today's interview for transcription but as stated in the Participant Information Sheet, you will not be identified in any publications and if you do mention anything that might identify you such, it will be *censored. Once the interview has been transcribed the recording will be deleted.*
We can take a break or stop at any time, you do not need to give me a reason for stopping and it will not impact on your relationship with any staff at [partner institution].
- *Are you okay with everything I just mentioned?*
- *Do you have any questions?*
- *Happy to proceed?*

Questions

1. Can you describe the current approach to wound management in [organisation]? What processes and practices are involved?

2. [Organisation] plans to implement a new digital wound model of care which includes use of a digital wound platform to monitor, measure, and document patient's wounds, both during and in-between appointments, as well as access to a to a specialist service for advice and shared care. What are your thoughts on this new model? Do you think it is needed?
    a. Prompt: Why? Why not?
    b. Prompt: What potential benefits or challenges do you foresee in adopting this new approach?

3. What are your thoughts on whether staff at [organisation] will adopt this new model of care?
    a. Prompt: How do you think they will engage with the digital wound platform? What factors might influence they willingness to use it?
    b. Prompt: How likely do you think they will utilise the wound specialist service? Why?

4. Based on your experience, do you think patients with wounds will be open to using a digital wound platform to monitor their wounds between face-to-face appointments? Why or why not?

    a. Prompt: What factors do you think might encourage them to participate?

5. In your opinion, who will be the key drivers of this model of care at [organisation]?
    a. Prompt: What actions or strategies do you think they will need to implement to effectively promote and drive this model forward?

6. How do you envision this model of care integrating with or complementing, existing work processes and practices in [organisation]?
    a. Prompt: What aspects do you think will align well, and are there any potential challenges you foresee?

7. What type of assistance and resources do you believe will be necessary to ensure the successful implementation of this model of care at [organisation]?

8. What specific steps do you think should be taken to facilitate our successful implementation of this model?

9. Do you have any other thoughts about this model of care or the implementation process?
    a. Prompt: Any additional insights or suggestions?
