# Supplementary material for: Implementing a digitally enabled wound model of care to improve chronic wound outcomes in seven health services in Australia: study protocol for a multisite, hybrid type 2, two-phase effectiveness-implementation trial (the ENABLE study)
Source: BMJ Open. 2026 Nov 3;16(11):e123987. doi: 10.1136/bmjopen-2026-123987 (PMC13735325; doi:10.1136/bmjopen-2026-123987)
Supplement: Supplementary file 11 [file bmjopen-16-11-s011.pdf]

Clinician Interview Guide – Post-Implementation of Digital Wound Model of Care

<u>*Introduction*</u>

Thank you for consenting to participate in this interview/focus group. My name is [name of interviewer] and I'm a [role of interviewer]. I am conducting research to understand your recent experience of the digital wound model of care implemented in your organisation. The model of care involved access to a specialist wound service and a digital wound platform. We will be exploring how it was implemented, what went well and what could be improved on. Your thoughts are very important to us to make sure we are meeting the needs of patients with chronic and complex wounds and ensure we can provide the best care for them. We plan to analyse and publish the results from these interviews.

- *Confirm the participant has read the Participant Information Sheet and answer any queries which arise*
- *Confirm they are still willing to participate*
- *Confirm the participant is happy for the interview to be audio-recorded*

I am recording today's interview for transcription but as stated in the Participant Information Sheet, you will not be identified in any publications and if you do mention anything that might identify you such, it will be *censored. Once the interview has been transcribed the recording will be deleted.*

We can take a break or stop at any time, you do not need to give me a reason for stopping and it will not impact on your relationship with any staff at [partner institution].

- *Are you okay with everything I just mentioned?*
- *Do you have any questions?*
- *Happy to proceed?*

Questions

1. To start, could you share your overall thoughts or initial impressions of the new model of care?
   a. Prompt: What stood out to you the most when you first began working with it?

2. In your experience, did the new model of care seem effective in your specific setting?

3. What factors contributed to its success or difficulties?
   a. Prompt: People, processes, organisation, intervention elements, workflows?

4. How did you and your colleagues respond to the new model?
   a. Prompt - How easy was it to get the team on board with the changes?

5. How flexible do you think the intervention is to accommodate the unique needs of your clinical environment?
   a. Prompt: Were there any aspects of the new model that felt incompatible with how you typically provide care? Which ones and why?

6. In your opinion, how well did the new model of care address the needs and concerns of the patients served by your organisation?

7. How did the patients respond to the new model of care?
    a. Prompt: Did you notice any changes in their outcomes or satisfaction?
    b. Prompt: Were there any particular aspects of the intervention that patients seemed to appreciate or struggle with?
    c. Prompt: What challenges or barriers did patients face when engaging with the new model of care?

8. Based on your experience, do you believe the intervention was effective in your setting?
    a. Prompt: Why or why not?

9. Once the intervention was in place, how confident were you in using it effectively in day-to-day patient care?

10. Is there anything else you would like to share regarding your thoughts on this model of care or the implementation process?
    a. Prompt: Any additional insights or suggestions?
