# Supplementary material for: Implementing a digitally enabled wound model of care to improve chronic wound outcomes in seven health services in Australia: study protocol for a multisite, hybrid type 2, two-phase effectiveness-implementation trial (the ENABLE study)
Source: BMJ Open. 2026 Nov 3;16(11):e123987. doi: 10.1136/bmjopen-2026-123987 (PMC13735325; doi:10.1136/bmjopen-2026-123987)
Supplement: Supplementary file 12 [file bmjopen-2026-123987.reviewer_comments.pdf]

PEER REVIEW HISTORY

BMJ Open publishes all reviews undertaken for accepted manuscripts. Reviewers are asked to complete a checklist review form and are provided with free text boxes to elaborate on their assessment. These free text comments are reproduced below.

ARTICLE DETAILS

Title (Provisional)

Implementing a digitally enabled wound model of care to improve chronic wound outcomes in seven health services in Australia: Study protocol for a multisite, hybrid type 2, two-phase effectiveness-implementation trial (the ENABLE study)

Authors

Barakat-Johnson, Michelle; Lai, Michelle; Wilson, Andrew; Howell, Martin; Stephenson, John; Chow, Josephine Sau Fan; Twigg, Stephen M; White , Kate; Frotjold, Astrid; Leahy, Catherine; Baysari, Melissa T

VERSION 1 - REVIEW

**Reviewer**	**1**
**Name**	**Sandoval, Cristian**
**Affiliation**	**Universidad de La Frontera, Faculty of Medicine**
**Date**	**02-Jul-2026**
**COI**	**None**

This is a well-written and timely protocol addressing an important healthcare challenge. The integration of implementation science with a stepped-wedge cluster randomized design is appropriate and the manuscript is generally comprehensive, methodologically sound, and consistent with SPIRIT recommendations. The study addresses a relevant evidence gap regarding the implementation of digitally enabled wound care across heterogeneous healthcare settings.

I have only a few suggestions that could further strengthen the protocol prior to publication:

* Although the primary outcome is clearly defined, additional detail regarding the planned statistical modelling of the stepped-wedge cluster randomized trial (including adjustment for secular trends, clustering effects, and handling of missing data) would improve reproducibility.

* Given that implementation fidelity is one of the secondary implementation outcomes, it would be helpful to describe in greater detail how fidelity will be measured, monitored and incorporated into the effectiveness analyses.

* The manuscript would benefit from a more explicit description of the AI-enabled wound assessment component, including whether algorithm performance will be monitored throughout the study and how clinicians will manage potential disagreements between AI recommendations and clinical judgement.

* Because participating sites differ substantially in digital infrastructure and workforce capacity, additional information describing strategies to minimize inter-site heterogeneity would strengthen the implementation component.

* A brief description of data governance, cybersecurity and management of patient-generated digital images would further reassure readers regarding privacy and ethical considerations.

Overall, this is a high-quality protocol describing an important multicentre implementation trial with strong potential clinical impact.

**Reviewer**	**2**
**Name**	**Ogrin, Rajna**
**Affiliation Institute**	**Bolton Clarke Support Centre, Bolton Clarke Research**
**Date**	**10-Jul-2026**
**COI**	**None**

Major Comment

This is a strong protocol describing a stepped-wedge cluster randomised evaluation of an important and timely wound care model. The study design, intervention description, stakeholder engagement and economic evaluation are notable strengths.

The manuscript is generally well developed from a clinical trial perspective. However, I found it difficult to determine the intended contribution of the implementation components of the study.

The protocol includes substantial emphasis on implementation outcomes, including acceptability, appropriateness, feasibility, fidelity and sustainability, yet implementation strategies, implementation mechanisms and implementation evaluation approaches are described in less detail than the effectiveness evaluation. This creates some ambiguity regarding whether implementation outcomes are intended primarily as exploratory or descriptive measures, or whether the study is also intended to make a substantive implementation science contribution.

I therefore encourage the authors to clarify whether the study is intended primarily as:

• a stepped-wedge effectiveness trial with embedded implementation outcomes; or

• an effectiveness–implementation hybrid study designed to generate implementation knowledge alongside effectiveness evidence.

This distinction is important because it influences the level of detail expected in relation to implementation strategies, implementation outcomes, process evaluation, fidelity and contextual factors.

Suggestions if implementation is a significant study objective

If the authors intend implementation to be a major component of the study, I believe the manuscript would benefit from additional information regarding:

1. The programme theory underpinning implementation

CFIR is appropriately identified as the guiding implementation framework. However, CFIR primarily assists in identifying determinants of implementation and does not itself explain how implementation activities are expected to influence implementation or clinical outcomes.

A brief implementation logic model, programme theory, or determinant–strategy–outcome mapping would help explain how implementation success is expected to be achieved.

2. The implementation strategy

Recognising that implementation strategies will emerge through the Phase 1 co-design process, it would nevertheless be useful to provide additional information regarding:

• how implementation strategies will be selected;

• who will deliver implementation support;

• who will receive implementation support;

• how implementation activities will be documented.

3. Implementation outcome measurement

The protocol would benefit from greater clarity regarding:

• implementation outcome definitions;

• data sources;

• measurement approaches;

• timing of assessment; and

• planned analyses.

Particular attention could be given to adoption, which appears less clearly articulated than the other implementation outcomes.

4. Process evaluation

The proposed qualitative work appears well suited to supporting a process evaluation.

Additional detail regarding:

• implementation questions to be explored;

• implementation mechanisms of interest;

• contextual influences; and

• reasons for variation across sites

would strengthen the explanatory value of the implementation component.

5. Fidelity, adaptation and context

Given the diversity of participating services, I encourage the authors to provide additional detail regarding:

• core intervention components;

• allowable adaptation;

• how adaptations will be documented; and

• how contextual influences may be captured during implementation.

6. Sustainability

As sustainability is identified as an implementation outcome, additional information regarding how sustainability will be assessed would be valuable.

Minor Comments

• Consider providing additional information regarding adoption as an implementation outcome.

• Consider clarifying site selection criteria and organisational readiness considerations.

• Consider including a table linking CFIR determinants, implementation activities and anticipated implementation outcomes.

• Clarify whether implementation costs will be distinguished from intervention costs within the economic evaluation.

VERSION 1 - AUTHOR RESPONSE

Reviewer 1: Dr Cristian Sandoval, Universidad de La Frontera

We thank Dr Sandoval for the positive assessment of the protocol and the constructive suggestions, each of which we have addressed below.

No.	Comment	Response	Page no. and section
R1.1	Although the primary outcome is clearly defined, additional	Thank you for your response.	p. 27, Data analysis,

No.	Comment	Response	Page no. and section
	detail regarding the planned statistical modelling of the stepped-wedge cluster randomized trial (including adjustment for secular trends, clustering effects, and handling of missing data) would improve reproducibility.	The Quantitative analysis section has been expanded.<br><br>*Primary outcome*<br><br>The primary outcome—number of hospital admissions for chronic or complex wounds—will be analysed using mixed-effects logistic regression, adjusted for important prognostic factors. *p*-values, odds ratios and 95% confidence intervals will be reported for treatment and control variables. Two levels of random effects will be included to account for clustering within sites and periods. The extent of data clustering will be assessed via determination of the variance partition coefficient of null models, and the model will be re-specified as necessary before the addition of covariates if the VPC indicates very low levels of residual variance associated with a particular level. This model formulation is robust to missing data, but excessive amounts of missing data will be assessed, and any cases or variables with >50% missing data will be removed from the analysis. Intervention status will be modelled using a binary fixed effect. Secular trends will be accounted for with the inclusion of a fixed categorical effect. Site-level and individual random effects will be included to account for within-site and within-individual variation. We will allow secular trends to vary across sites via the inclusion of a time-cluster interaction term.	Quantitative analysis, Primary outcome

No.	Comment	Response	Page no. and section
		Model goodness-of-fit, linearity, calibration, classification and discrimination will be assessed, and residual analysis will be conducted to identify potentially influential data points.	
R1.2	Given that implementation fidelity is one of the secondary implementation outcomes, it would be helpful to describe in greater detail how fidelity will be measured, monitored and incorporated into the effectiveness analyses.	A description of fidelity measurement has been added.<br><br>Fidelity is defined as delivery of the core components of the ENABLE model as intended (specialist review via the Wound Care Command Centre, digital wound assessment using the Tissue Analytics app, and documented shared-care communication).<br>It will be measured using app usage analytics (proportion of enrolled patients with wound assessments captured digitally), training completion records, wound consults under the new model of care each cluster, conducted during the intervention period.<br>Fidelity data will be reviewed by the clinical expert and project steering group with feedback provided to sites as part of the monitoring and feedback strategy. In the effectiveness analyses, fidelity will be reported descriptively by cluster and used in a pre-specified sensitivity analysis restricted to clusters and periods meeting a minimum fidelity threshold, with the intention-to-treat analysis remaining primary. Qualitative interviews with clinicians and patients will further explore factors influencing fidelity.	p. 16, Implementation strategy; p. 21 Outcomes; Data analysis
R1.3	The manuscript would benefit from a more explicit description of the AI-enabled wound assessment component, including whether algorithm performance will be monitored throughout the study and how clinicians will	We thank the reviewer for this comment. Tissue Analytics has been used at Sydney Local Health District for seven years, with robust governance, iterative refinement and sustained industry partnership - all documented in our previous studies (references 17, 18 and 19). The app's	p. 15, Intervention

No.	Comment	Response	Page no. and section
	manage potential disagreements between AI recommendations and clinical judgement.	performance and clinical integration are therefore well established prior to this trial.<br><br>Clinicians receive training on the app as part of the ENABLE implementation strategy and are explicitly instructed to apply their clinical judgement at all times. Where a clinician considers an app-generated measurement to be inaccurate, they have the option to retrace the wound boundary and recapture the measurement. Treatment decisions rest entirely with the treating clinician.<br><br>We also note that algorithm performance monitoring is not an objective of this study. The ENABLE study evaluates the scalability and real-world implementation of a digitally enabled model of care that has already been shown to be feasible and acceptable in our earlier work (references 17, 18 and 19). The focus here is on how that model performs across diverse healthcare settings, not on the technical performance of the underlying app.<br><br><u>The manuscript has nonetheless been updated to clarify the decision-support role of the app, the clinician override process and the study's alignment</u> with the Australian National Framework for the Assurance of Artificial Intelligence in Government and the NSW Artificial Intelligence Assessment Framework (references 36 and 37).<br><br>The app functions as a decision-support tool; all treatment decisions rest with the treating clinician. Where a clinician considers an app-generated measurement to be inaccurate, they may retrace the wound boundary and recapture the measurement. The study aligns with the Australian National Framework for the Assurance of Artificial Intelligence in Government and the NSW Artificial Intelligence Assessment Framework.[37,38]	
R1.4	Because participating sites differ substantially in digital infrastructure and workforce	We have added detail on how heterogeneity is managed.	p. 17, Context and settings;

No.	Comment	Response	Page no. and section
	capacity, additional information describing strategies to minimize inter-site heterogeneity would strengthen the implementation component.	First, a structured site readiness assessment (digital infrastructure, workforce capacity and leadership support) will be completed for each site before enrolment, and sites within larger services were selected on this basis. Second, all sites will receive standardised training on the ENABLE model and the Tissue Analytics app, delivered by local champions appointed at each site to support uptake. Third, the Phase 1 co-design process deliberately tailors implementation strategies to each cluster's context while preserving the core components of the model, so that heterogeneity is addressed through planned adaptation. Finally, monthly cross-site meetings of the clinical expert and consumer group will provide a mechanism for identifying and responding to emerging site-level barriers during rollout.	Implementation strategy
R1.5	A brief description of data governance, cybersecurity and management of patient-generated digital images would further reassure readers regarding privacy and ethical considerations.	A data governance paragraph has been added to the Data management section.<br><br>Wound images captured in the Tissue Analytics app are stored within the app's secure, encrypted environment and linked to the patient's electronic medical record; images are not stored on clinicians' personal devices. Access is role-based and restricted to the treating team and authorised study personnel. The platform is TGA-listed (ARTG ID 374066) and its deployment complies with NSW Health data security and privacy requirements, applicable privacy legislation, and the NSW Artificial Intelligence Assessment Framework.[38] Study data are held on University of Sydney secure systems as described in the Data management section, with de-identification applied before analysis and data sharing.	p. 23, Data management; Intervention

Reviewer 2: Dr Rajna Ogrin, Bolton Clarke Support Centre

We thank Dr Ogrin for this thoughtful review, which has materially strengthened the implementation component of the protocol.

No.	Comment	Response	Page no. and section
R2.0	Major comment: I found it difficult to determine the intended contribution of the implementation components of the study... I therefore encourage the authors to clarify whether the study is intended primarily as: a stepped-wedge effectiveness trial with embedded implementation outcomes; or an effectiveness-implementation hybrid study designed to generate implementation knowledge alongside effectiveness evidence.	Thank you for prompting this clarification. The ENABLE study is a hybrid type 2 effectiveness-implementation study: it is designed to generate implementation knowledge alongside effectiveness evidence, with dual and co-equal aims.<br>This is reflected in the study objectives (implementation evaluation is objective 1), the CFIR-guided co-design of implementation strategies in Phase 1, and the pre-specified implementation outcomes.<br><br>We have now stated this explicitly in the title, Abstract, Introduction and Study design section, and have strengthened the implementation methods throughout in line with the reviewer's specific suggestions (points 1-6 below).<br><br>In classifying the study we were guided by Fortney et al. (2024), who distinguish hybrid trials from pragmatic effectiveness trials on the basis that hybrid type 2 studies evaluate a clinical intervention and practical, replicable implementation strategies concurrently, with implementation outcomes specified a priori.	Abstract; Introduction p. 4; Study design p.11
R2.1	The programme theory underpinning implementation. CFIR is appropriately identified as the guiding implementation framework. However, CFIR primarily assists in identifying determinants of implementation and does not itself explain how implementation activities are expected to influence implementation or clinical outcomes. A brief implementation logic model,	Agreed. We have added an implementation logic model as a new figure (Figure [5]) and accompanying text.<br><br>Finally, monthly cross-site meetings of the clinical expert and consumer group will provide a mechanism for identifying and responding to emerging site-level barriers during rollout.<br><br>Figure 5 summarises the full implementation logic model, integrating these strategies with the anticipated	p. 19, Implementation strategy; new Figure [5]

No.	Comment	Response	Page no. and section
	programme theory, or determinant-strategy-outcome mapping would help explain how implementation success is expected to be achieved.	determinants, mechanisms and outcomes across all participating clusters.<br><br>**Figure 5. ENABLE implementation logic model.** [At end of this table]<br><br>The model maps: (i) CFIR determinants across five domains (innovation, inner setting, outer setting, individuals and implementation process); (ii) tailored implementation strategies selected to address them; (iii) the mechanisms through which strategies are expected to operate; (iv) anticipated implementation outcomes (acceptability, appropriateness, feasibility, adoption, fidelity and sustainability); and (v) downstream clinical and service outcomes (wound healing, reduced hospitalisations and improved continuity of care). This mapping reflects the hybrid type 2 design of the study, in which implementation strategies are specified a priori alongside effectiveness aims. Developed with reference to the CFIR Outcomes.	
R2.2	The implementation strategy. Recognising that implementation strategies will emerge through the Phase 1 co-design process, it would nevertheless be useful to provide additional information regarding: how implementation strategies will be selected; who will deliver implementation support; who will receive implementation support; how implementation activities will be documented.	This detail has been added to the Implementation strategy section.<br><br>Selection: candidate strategies are generated by mapping the CFIR-coded barriers and facilitators from the Phase 1 qualitative analysis to strategies, which are then prioritised and operationalised for each cluster by the clinical expert and consumer group on the basis of feasibility, local fit and anticipated impact. Delivery: implementation support is delivered by cluster research officers and site-based local champions, supported by the central project team and, for clinical practice questions, the Wound Care Command Centre specialists.<br><br>Recipients: implementation support is directed at the clinicians delivering wound care at each site (as listed in Table 2) and	p. 17, Implementation strategy

No.	Comment	Response	Page no. and section
		their line managers, whose engagement is required for workflow change.<br>Documentation: all implementation activities (training sessions, site meetings, newsletters, feedback reports and any adaptations) are recorded prospectively in a structured implementation log maintained by each cluster research officer, which will be used in the process evaluation and fidelity assessment.	
R2.3	Implementation outcome measurement. The protocol would benefit from greater clarity regarding: implementation outcome definitions; data sources; measurement approaches; timing of assessment; and planned analyses. Particular attention could be given to adoption, which appears less clearly articulated than the other implementation outcomes.	A new table (Table [4]) has been added defining each implementation outcome, its data source, measurement approach, timing and planned analysis, using the definitions consolidated in Proctor et al.'s (2023) scoping review of implementation outcomes.<br>In brief: acceptability, appropriateness and feasibility are assessed through clinician surveys and CFIR-guided interviews and focus groups during Phase 2; adoption is now explicitly defined and measured as (i) the proportion of eligible clinicians at each site who complete training and actively use the ENABLE model, and (ii) the proportion of eligible patients at each site managed through the model during the intervention period, both derived from training records and app and medical record data; fidelity is measured as described in R1.2 above; and sustainability is addressed in R2.6 below. Quantitative implementation outcomes will be summarised descriptively by cluster and period; qualitative data will be analysed thematically against the CFIR as described in the Data analysis section.	p. 17, Outcomes; new Table [4]; Data analysis
R2.4	Process evaluation. The proposed qualitative work appears well suited to supporting a process evaluation. Additional detail regarding: implementation questions to be explored; implementation mechanisms of interest; contextual influences; and reasons for variation across sites would	We have reframed the Phase 2 qualitative and implementation-monitoring work explicitly as a process evaluation and added the questions it will address<br><br>*Patient and clinician interviews*<br><br>Semi-structured interviews and focus groups will be conducted with patients and clinicians in both Phase 1 and Phase 2 to explore their experiences of wound care	p. 23, Study design; Data collection; Data analysis p.25

No.	Comment	Response	Page no. and section
	strengthen the explanatory value of the implementation component.	and the implementation of the ENABLE model. Patients will be invited by the cluster research officer to be interviewed on their experiences with usual care (Phase 1) and the ENABLE intervention (Phase 2). Clinicians will participate in interviews or focus groups examining their current practices and barriers (Phase 1) and model implementation outcomes, including feasibility, sustainability and facilitators and barriers (Phase 2), guided by the CFIR.<br><br>The Phase 2 qualitative data collection, implementation logs and quantitative implementation outcome data will together constitute a process evaluation. The full patient and clinician interview and focus group guides for both phases are provided in Online Supplemental Appendices 8-11].<br><br>*Qualitative analysis*<br><br>Braun and Clarke's[44] six-step thematic analysis, informed by the CFIR, will be used to understand the barriers and facilitators to implementation and potential solutions to the barriers. Two authors (AF and ML) will independently review each transcript and extract themes using a general inductive approach. A third author (MB) will review the analyses and arbitrate discrepancies where necessary to reach consensus. Emerging themes will then be mapped to the CFIR domains and constructs. The researchers will meet periodically throughout the data analysis to discuss the themes, resolve discrepancies and reach a consensus on barriers, facilitators and proposed solutions. In Phase 2, qualitative findings will be integrated with implementation log data and quantitative implementation outcomes to address the process evaluation questions, with cross-cluster comparison used to explain variation in implementation and outcomes across diverse settings. The findings will inform the identification of barriers, facilitators and strategies to	

No.	Comment	Response	Page no. and section
		support the large-scale implementation and sustainability of ENABLE.<br><br>These questions are embedded in the Phase 2 interview and focus group guides (now provided as supplementary appendices) and will be analysed alongside the implementation logs and quantitative implementation outcome data, with cross-cluster comparison used to explain variation.	
R2.5	Fidelity, adaptation and context. Given the diversity of participating services, I encourage the authors to provide additional detail regarding: core intervention components; allowable adaptation; how adaptations will be documented; and how contextual influences may be captured during implementation.	Thank you for this comment. The Intervention and Implementation strategy sections have been revised to distinguish the core components of the ENABLE model from its adaptable periphery.<br><br>Intervention<br>The core components of the ENABLE model, required at all sites, are: access to specialist wound review through the Wound Care Command Centre; digital wound assessment and monitoring using the Tissue Analytics app; and documented shared-care communication between the specialist service and the local treating team. Adaptable elements, which may be tailored to local context, include referral and escalation pathways, the mix of virtual and face-to-face review, the frequency of review (which is clinically determined), local workflow integration and the format of training and communication.<br><br>Implementation strategy<br><br>End of documentation section:<br>Adaptations will be recorded prospectively in the implementation log, including what was adapted, by whom, why and with what anticipated effect, and will be reviewed by the clinical expert and consumer group to confirm that core components remain intact.	p. 15,16 Intervention; Implementation strategy p.17
R2.6	Sustainability. As sustainability is identified as an implementation outcome,	A description of sustainability assessment has been added.	p. 20, Outcomes; Data collection;

No.	Comment	Response	Page no. and section
	additional information regarding how sustainability will be assessed would be valuable.	Implementation outcomes, last dot point:<br>Sustainability will be assessed in two ways: perceived sustainability, through clinician interviews and focus groups in the final 2 months of each cluster's intervention period exploring intentions and perceived capacity to continue the model and the organisational conditions required; and continued delivery, through routinely collected app and service data describing use of the model at each site for up to 12 months after site completion, reported descriptively.<br><br>Expected outcomes:<br><br>Consistent with the implementation-focused nature of the study, we also expect to identify key barriers and facilitators influencing adoption, fidelity and sustainability of the ENABLE model across participating sites. These findings will be used to refine the intervention and implementation strategy, ensuring adaptability to local contexts while maintaining core model integrity. It is anticipated that clusters with higher baseline digital maturity and stronger interdisciplinary integration will demonstrate more rapid and sustained uptake of the ENABLE model. Sustainability findings will inform the translation consortium's scale-up and sustainability planning with NSW Health partners.	Expected outcomes p.30
R2.m1	Minor: Consider providing additional information regarding adoption as an implementation outcome.	Addressed in R2.3 - adoption is now explicitly defined, with data sources, measurement and timing specified in the new implementation outcomes table, and has been added to the implementation outcome lists in the Abstract and Outcomes section (and the registry; see E9).	Abstract; Outcomes; new Table [4]
R2.m2	Minor: Consider clarifying site selection criteria and	The Context and settings section now states the site selection criteria:	p. 8, Context and settings

No.	Comment	Response	Page no. and section
	organisational readiness considerations.	For partner services comprising multiple facilities, sites were selected on the basis of a structured readiness assessment covering feasibility of trial procedures, workforce capacity to deliver the model, digital infrastructure, and executive and clinical leadership support for implementation.	
R2.m3	Minor: Consider including a table linking CFIR determinants, implementation activities and anticipated implementation outcomes.	Agreed.<br>This mapping is provided in the new implementation logic model (Figure 5)	p. 19, new Figure [5]; new Online
R2.m4	Minor: Clarify whether implementation costs will be distinguished from intervention costs within the economic evaluation.	Thank you we have updated the economic evaluation section *Economic evaluation*<br><br>A within-trial cost-effectiveness and cost–utility analysis comparing intervention and standard care groups will be undertaken from the perspective of the public healthcare funder.<br><br>Healthcare costs will be estimated by applying national weighted activity units and the national efficient price to activity-based data. Costs for non-hospital services will be derived from Medicare Benefits Schedule and Pharmaceutical Benefits Scheme datasets. Implementation costs (training, implementation support time, site onboarding and adaptation activities, drawn from the implementation logs) will be identified and reported separately from intervention delivery costs (specialist service time, app licensing and clinical consumables); research-related expenses will be excluded. Both cost categories will be included in the base-case analysis, with a sensitivity analysis excluding one-off implementation costs to reflect steady-state delivery. Analyses will account for variations in service delivery and funding models by estimating costs separately by clinic type.	p. 28, Economic evaluation

New references added to the manuscript

1. Damschroder LJ, Reardon CM, Opra Widerquist MA, Lowery J. Conceptualizing outcomes for use with the Consolidated Framework for Implementation Research (CFIR): the CFIR Outcomes Addendum. Implement Sci. 2022 Jan 22;17(1):7. doi: 10.1186/s13012-021-01181-5.
2. Proctor EK, Bunger AC, Lengnick-Hall R, Gerke DR, Martin JK, Phillips RJ, Swanson JC. Ten years of implementation outcomes research: a scoping review. *Implement Sci.* 2023;18(1):31. doi:10.1186/s13012-023-01286-z
3. Fortney JC, Curran GM, Lyon AR, Check DK, Flum DR. Similarities and Differences Between Pragmatic Trials and Hybrid Effectiveness-Implementation Trials. J Gen Intern Med. 2024 Jul;39(9):1735-1743. doi: 10.1007/s11606-024-08747-1. Epub 2024 Apr 16. PMID: 38627320; PMCID: PMC11254859.

VERSION 2 - REVIEW

**Reviewer**	**1**
**Name**	**Sandoval, Cristian**
**Affiliation**	**Universidad de La Frontera, Faculty of Medicine**
**Date**	**22-Aug-2026**
**COI**	

Thank you for have satisfactorily addressed my previous comments. The revised manuscript has improved substantially in methodological clarity and reproducibility. In particular, the expanded description of the statistical analysis, implementation fidelity assessment, AI-enabled wound assessment component, management of inter-site heterogeneity, and data governance adequately addresses the issues raised in my initial review.

I have only one minor editorial observation. The primary outcome is defined elsewhere in the manuscript as the proportion of patients admitted to hospital with a chronic or complex wound, whereas the Quantitative Analysis section refers to the “number of hospital admissions.” Given that the proposed analysis uses mixed-effects logistic regression and the sample size calculation is based on the proportion of patients admitted, I suggest harmonising this terminology throughout the manuscript to clearly indicate that the primary outcome is binary at the patient level.

**Reviewer**	**2**
**Name**	**Ogrin, Rajna**
**Affiliation**	**Bolton Clarke Support Centre, Bolton Clarke Research Institute**
**Date**	**10-Aug-2026**
**COI**	

Overall assessment

The authors have responded comprehensively to my comments and have substantially strengthened the implementation science components of the protocol. In particular, the study is now clearly positioned as a Hybrid Type 2 effectiveness–implementation study, with explicit implementation aims, a logic model, a detailed implementation strategy description, operationalisation of implementation outcomes, a process evaluation framework, fidelity assessment, adaptation tracking, sustainability assessment and clarification of implementation costs. These revisions address the major concerns raised in my original review and considerably improve the transparency and reproducibility of the implementation evaluation. Remaining issues from my original comments are relatively minor, specifically, the following 2 areas could be improved:

1. Mixed-methods integration

I had suggested stronger description of how qualitative and quantitative findings would be integrated. You now state that qualitative findings will be integrated with implementation logs and implementation outcomes and cross-cluster comparisons will be used.

However you still do not explicitly describe whether you will use: triangulation; joint displays or integration procedures for a mixed-methods audience. This could be clarified.

2. Context monitoring

The revised manuscript improves contextual reporting and readiness assessment substantially. However, there is still no explicit context monitoring framework throughout implementation. This could be articulated.

Other areas that would benefit from improvement:

1. Hybrid Type 2 designation is now stated, but the implementation outcomes are still not truly "co-equal"

As the study is now positioned as a Hybrid Type 2 effectiveness-implementation trial, consider clarifying whether any implementation outcomes are considered primary implementation endpoints, or whether implementation outcomes are exploratory and descriptive in nature.

2. The implementation logic model is helpful but still fairly generic

The new logic model is a major improvement. However, it's more of a conceptual implementation science figure than a study-specific theory. For example, determinants are broad; mechanisms remain generic; and relationships between implementation and clinical outcomes are implied rather than explicit.

For a protocol this is acceptable, but I do note that the figure will be most useful if the final trial publications revisit these proposed mechanisms and report whether the hypothesised pathways were supported.

3. Sustainability wording contains a small inconsistency

In the outcomes section you write “continued use for up to 12 months after site completion, but in Table 4 it is written: “continued use for up to 6 months after site completion.“

This should be corrected.

4. There are now some duplicated sustainability statements

The sustainability section appears to repeat similar content more than once in the outcomes section. A little editorial tightening would improve readability.

VERSION 2 - AUTHOR RESPONSE

Reviewer 1 (Dr Cristian Sandoval)

No.	Comment	Response	Page no. and section
R1.1	The primary outcome is defined elsewhere as the proportion of patients admitted to hospital with a chronic or complex wound, whereas the Quantitative Analysis section refers to "number of hospital admissions". Suggest harmonising terminology to clearly indicate the primary outcome is binary at the patient level.	Thank you - we agree this was inconsistent. The Quantitative Analysis section has been amended to describe the primary outcome as "the proportion of patients admitted to hospital with a chronic or complex wound", consistent with its definition in the Outcomes section and with the binary, patient-level mixed-effects logistic regression model and sample size calculation.	p.25 Data analysis, Quantitative analysis, Primary outcome

Reviewer 2 (Dr Rajna Ogrin)

No.	Comment	Response	Page no. and section
R2.1	Mixed-methods integration: it is stated that qualitative findings will be integrated with implementation logs and quantitative outcomes, with cross-cluster comparison used to explain variation, but no named integration procedure (e.g. triangulation, joint displays) is described.	We have added that “integration will follow a triangulation protocol, with qualitative themes, implementation log data and quantitative implementation outcomes brought together in joint display matrices by cluster, to identify areas of convergence, divergence and complementarity across data sources.”	p.25<br>Data analysis, Qualitative analysis
R2.2	Context monitoring: contextual reporting and readiness assessment have improved, but there is no explicit framework for	We have added a statement that “Contextual change will be monitored prospectively throughout implementation via the structured implementation log (recording	p.17 Implementation strategy (following the monthly cross-site meetings sentence)

No.	Comment	Response	Page no. and section
	monitoring context throughout implementation.	changes in staffing, workflow or digital infrastructure at each site) together with CFIR-guided discussion of emerging contextual factors at the monthly cross-site meetings, providing a running record for use in the process evaluation."	
R2.3	Hybrid Type 2 designation is now stated, but it is unclear whether any implementation outcomes are primary implementation endpoints or whether all are exploratory/descriptive.	We have added a sentence clarifying that all implementation outcomes in Table 4 are treated as co-primary implementation endpoints, assessed descriptively and thematically rather than through formal hypothesis testing, consistent with a hybrid type 2 design in which implementation and effectiveness aims are addressed concurrently.<br>"All implementation outcomes in Table 4 are treated as co-primary implementation endpoints, assessed descriptively and thematically rather than through formal hypothesis testing, consistent with a hybrid type 2 design in which implementation and effectiveness aims are addressed concurrently."	p.11 Study design (hybrid type 2 paragraph)
R2.4	The implementation logic model is a helpful improvement but remains fairly generic (broad determinants, generic mechanisms, implied rather than explicit relationships to clinical outcomes); acceptable for a protocol, but the reviewer notes it should be revisited in the final trial publications.	We thank Reviewer 2 for this observation and agree. We have added a sentence noting that "The hypothesised pathways in the logic model will be revisited and reported against in the trial's primary effectiveness and process evaluation publications."	p. 18 Figure 5 legend / segue sentence
R2.5	Sustainability wording is inconsistent: the outcomes text states continued use is assessed "for up to 12 months after site completion", whereas Table 4 states "up to 6 months after site completion".	Thank you for catching this. We have standardised this to six months after site completion throughout, matching Table 4	p.20<br>Secondary outcomes (Implementation outcomes/Sustainability) and Table 4

No.	Comment	Response	Page no. and section
R2.6	The sustainability section appears to repeat similar content more than once in the outcomes section; some editorial tightening would improve readability.	We have removed the duplicated sustainability text and retained a single, consolidated statement describing both the perceived-sustainability (clinician interview) and continued-delivery (app and service data) components.	p.20<br>Secondary outcomes (Implementation outcomes/Sustainability)

VERSION 3 - REVIEW

Reviewer 2

Name Ogrin, Rajna

Affiliation Bolton Clarke Support Centre, Bolton Clarke Research **Institute**

Date 11-Sep-2026

COI

Thank you for addressing my comments. This is a strong study and I look forward to reading your future publications.
